# Translation of Chemical Structure into Dissipative Particle Dynamics Parameters for Simulation of Surfactant Self-Assembly

**DOI:** 10.1021/acs.jpcb.1c00480

**Published:** 2021-04-13

**Authors:** Ennio Lavagnini, Joanne L. Cook, Patrick B. Warren, Christopher A. Hunter

**Affiliations:** † Department of Chemistry, University of Cambridge, Lensfield Road, Cambridge CB2 1EW, U. K.; ‡ Unilever R&D Port Sunlight, Quarry Road East, Bebington CH63 3JW, U. K.; § The Hartree Centre, STFC Daresbury Laboratory, Warrington WA4 4AD, U. K.

## Abstract

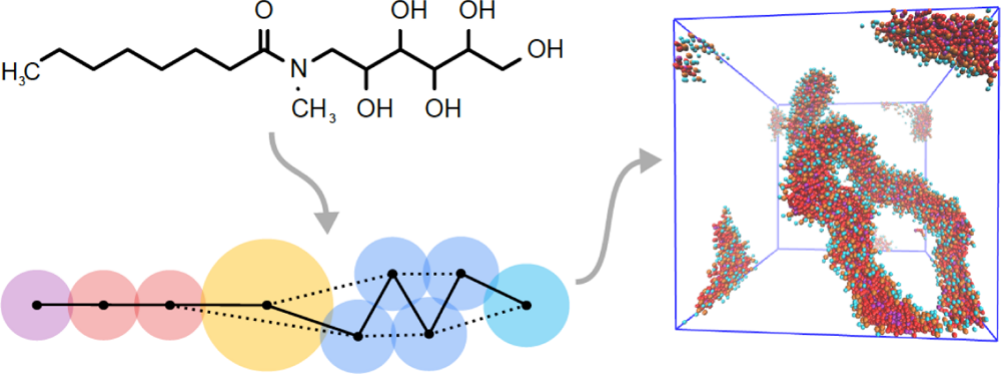

Dissipative particle dynamics (DPD) can be used to simulate the self-assembly properties of surfactants in aqueous solutions, but in order to simulate a new compound, a large number of new parameters are required. New methods for the calculation of reliable DPD parameters directly from chemical structure are described, allowing the DPD approach to be applied to a much wider range of organic compounds. The parameters required to describe the bonded interactions between DPD beads were calculated from molecular mechanics structures. The parameters required to describe the nonbonded interactions were calculated from surface site interaction point (SSIP) descriptions of molecular fragments that represent individual beads. The SSIPs were obtained from molecular electrostatic potential surfaces calculated using density functional theory and used in the SSIMPLE algorithm to calculate transfer free energies between different bead liquids. This approach was used to calculate DPD parameters for a range of different types of surfactants, which include ester, amide, and sugar moieties. The parameters were used to simulate the self-assembly properties in aqueous solutions, and comparison of the results for 27 surfactants with the available experimental data shows that these DPD simulations accurately predict critical micelle concentrations, aggregation numbers, and the shapes of the supramolecular assemblies formed. The methods described here provide a general approach to determining DPD parameters for neutral organic compounds of arbitrary structure.

## Introduction

Molecular modeling is a powerful tool for investigating molecular self-assembly in the liquid phase.^1,2^ These calculations can provide information at the molecular level about how intermolecular interactions affect macroscopic properties. With the increase in computational power and the availability of efficient parallel libraries,^3^ all-atom molecular dynamics (MD) simulations have become the method of choice for many computational investigations.^4−6^ However, MD simulations of the self-assembly of multicomponent systems require length and time scales beyond what is available in most standard research facilities. In these cases, coarse graining (CG) approaches can be useful. Bespoke force fields, such as MARTINI, have been successfully used in CG–MD simulations of surfactant aggregation in aqueous solutions.^7−10^ An alternative CG approach is dissipative particle dynamics (DPD), which is based on soft repulsive interactions that give improved scaling compared with hard-sphere approaches.^11^ Here, we apply the DPD method to the simulation of surfactant aggregation in aqueous solutions for a broad range of different compounds and develop a generalized computational approach to obtaining the required parameters based on the chemical structure.

In DPD, the overall force acting on a single bead is divided into three contributions, the conservative, drag, and random forces. The drag and random forces are used for thermostatic reasons and are correlated.^12^ The conservative force accounts for the nonbonded interactions between beads: for two beads *i* and *j*, the short-range interaction potential has the form
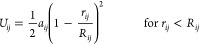
1where *a*_*ij*_ is the repulsion parameter describing the interaction energy associated with a contact between bead *i* and bead *j*, *r*_*ij*_ is the distance between bead centers, and *R*_*ij*_ is the limit beyond which the interaction becomes null, which is related to the effective radii of the beads (values of *R*_*ij*_ are obtained from the partial volume calculation method developed by Zipper and Durchschlag).^13−17^

In addition, bond length and bond angle potentials are used to describe interactions between bonded beads. The distance between two covalently bonded beads is described using a harmonic spring potential (eq 2), and chain rigidity is provided by a 1–3 harmonic bond angle potential (eq 3).
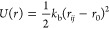
2where *k*_b_ is the spring constant (150 *k*_B_*T* was shown previously to be an appropriate value)^16^ and *r*_0_ is the equilibrium distance.
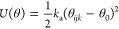
3where *k*_a_ is the spring constant (5 *k*_B_*T* was shown previously to be an appropriate value),^16^ θ_*ijk*_ is the angle formed by the three beads, and θ_0_ is the equilibrium angle.

The key nonbonded parameters that relate the outcome of a DPD simulation to the chemical structure are the repulsion parameters *a*_*ij*_, which are obtained from eqs 4 and 5.^18^
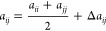
4where *a*_*ii*_ and *a*_*jj*_ are the self-interaction parameters and Δ*a*_*ij*_ describes the difference in the interactions between different types of beads.
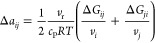
5where *c*_P_ is the matching constant that converts the energy into DPD units (a value of 0.291 has been shown to be appropriate for the bead density of 3 used here),^20^*v*_r_ is the volume of a water bead, *v*_*i*_ and *v*_*j*_ are the volumes of beads *i* and *j*, respectively, and Δ*G*_*ij*_ is the change in free energy for the transfer of bead *i* from the pure liquid to a dilute solution in bead *j* and vice versa for Δ*G*_*ji*_.^18^

The self-interaction parameters can be obtained by matching simulations to experimental liquid densities using a method introduced by Anderson *et al.*(16) The transfer free energies required in eq 5 can be estimated by using values for the mixing of liquids that most closely approximate the chemical structures of the relevant beads.^18,19^ A number of different approaches to deriving *a*_*ij*_ repulsion parameters have been described using experimental or calculated transfer free energies.^14,18−20^ Groot and Warren matched the equilibrium distance with the maximum in the radial distribution function to develop a soft sphere model for linear polymers (*a*_*ij*_ = 25 and ρ = 3).^20^ Since then, it has become common practice to choose values depending on the situation requirements.^15,21−27^

The key bonded parameters that relate the outcome of a DPD simulation to the chemical structure are the equilibrium distances and angles, *r*_0_ and θ_0_. Milano and Muller–Plathe introduced a systematic procedure for parameterization of bond distances using MD.^28−31^ Ortiz *et al.* proposed a similar approach specifically for DPD simulations,^32^ and Vishnyakov *et al.* developed parameters for chain rigidity and equilibrium bond distances based on MD simulations.^33^ Due to the nonbonded repulsion between bonded beads, the average bond distance obtained during a DPD simulation differs from the value of *r*_0_ in the bond potential (eq 2). Anderson *et al.* have shown that the number of heavy atoms in a bead can be used to estimate values of *r*_0_ for linear molecules.^16^ The equilibrium distance between two ethylene units in an alkyl chain is 2.52 Å, which corresponds to 0.445*r*_c_ in DPD units. To obtain this result in a simulation, the value of *r*_0_ must be set to 0.390*r*_c_. For bonds between beads with a different number of heavy atoms, the value of *r*_0_ was adjusted by 0.1*r*_c_ for each heavy atom.

Here, we develop a general approach that can be used to obtain both the bonded and nonbonded parameters required for DPD simulations directly from molecular mechanics and quantum chemical calculations on the molecule of interest. The approach has been tested on 27 different surfactants, and the DPD simulations are shown to provide an excellent description of the critical micelle concentration (CMC), the aggregation number (*N*_agg_), and aggregate shape when compared with experimental data.

## Results and Discussion

**Figure 1:**
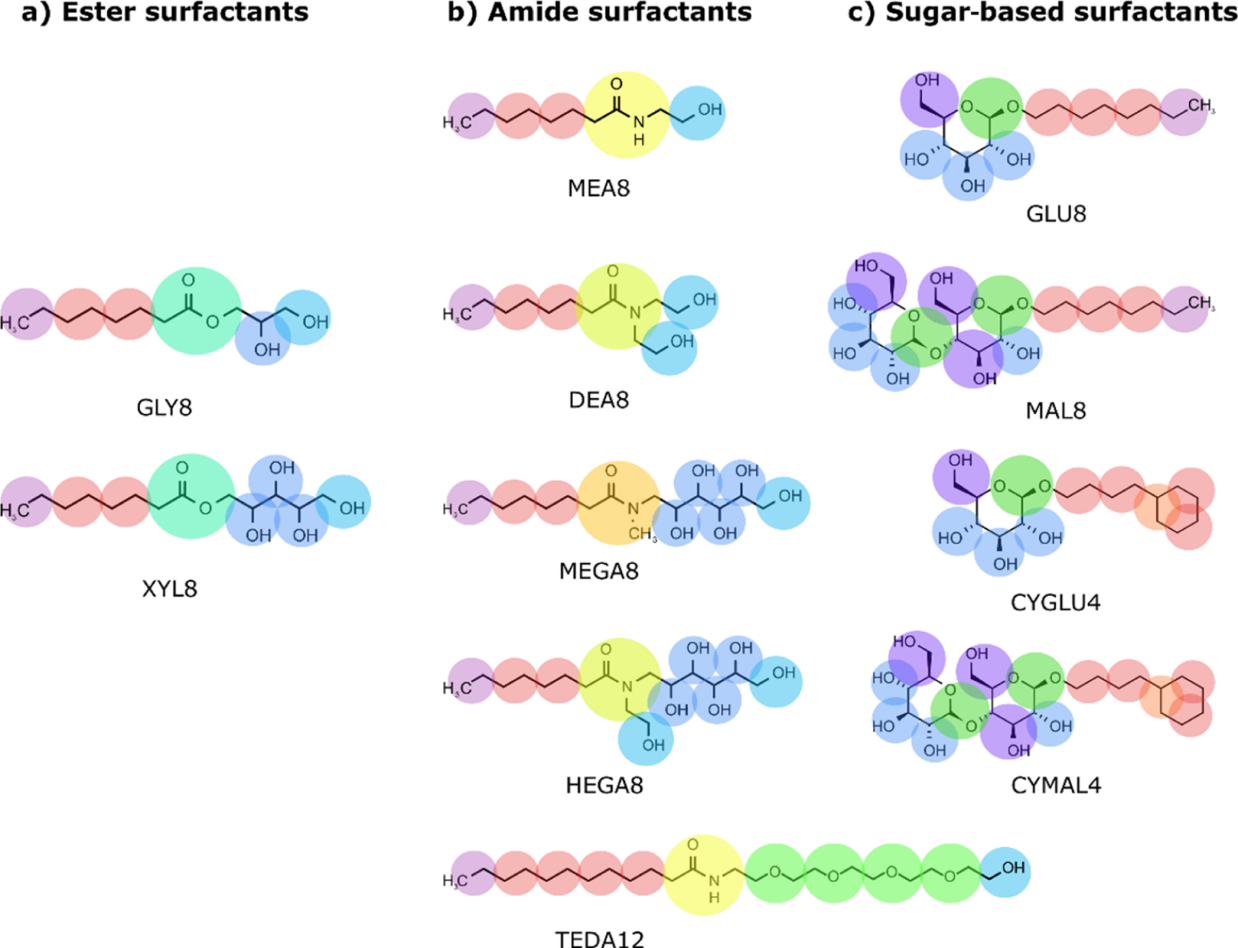
Coarse-grained representations of (a) ester surfactants, (b) amide surfactants, and (c) sugar surfactants.

In order to expand the range of surfactants that can be described using DPD, we have developed parameters for the 27 compounds illustrated in Figure 1. Figure 1 shows the way in which these compounds are coarse-grained as a set of DPD beads and highlights a number of new bead types for which DPD repulsion parameters are not currently available. The beads range in size from a single heavy atom (the purple beads used to describe terminal methyl groups in Figure 1) to beads containing six heavy atoms, which are required for tertiary amides (yellow beads). The surfactants in Figure 1 contain a range of different chemical functionalities, different sizes and shapes of a polar head group, and different hydrophobic chain lengths, providing a good test of how well DPD describes the relationship between surfactant properties and chemical structures.

### DPD Repulsion Parameters

The nonbonded repulsion parameters, *a*_*ij*_, were obtained from the values of Δ*G*_*ij*_, which were calculated using the SSIMPLE algorithm as described previously.^13^ In this method, each DPD bead is described as a set of surface site interaction points (SSIPs), each of which corresponds to 9 Å^2^ of the van der Waals surface and is assigned an interaction parameter ε based on polarity. The solvation energy of a bead in a liquid is based on pairwise interactions between SSIPs. Thus, a liquid is described as a collection of interacting SSIPs, and the equilibrium constant for the interaction between two SSIPs *x* and *y* is given by eq 6.^34^
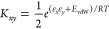
6where ε_*x*_ and ε_*y*_ represent the polarities of the two SSIPs and *E*_vdW_ is the van der Waals interaction energy between two SSIPs, which has a constant value of −5.6 kJ mol^–1^ based on experimental data on vapor–liquid equilibria of nonpolar liquids.

The concentrations of the SSIPs and the equilibrium constants for all pairwise interactions are used to determine the speciation of SSIP interactions in the liquid. For SSIP *x*, the fraction that does not interact with any other SSIP, *x*_f_, allows the chemical potential in different phases to be related. Equation 7 gives the solvation free energy of SSIP *x* in a liquid of bead *i*, Δ*G*_*x*_(*i*). The first term describes the interactions made with the other SSIPs in the liquid by using the fraction of free SSIPs. The second term accounts for the confinement of the SSIPs in a condensed phase and is obtained by using an equilibrium constant of unity in place of eq 6 to calculate the fraction of free SSIPs in a phase of the same SSIP concentration where there are no interactions.^13^
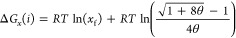
7where θ is the fractional SSIP occupancy relative to the maximum possible SSIP concentration of 300 M.

The free energy of transfer of a bead from one liquid to another is then given by summing the solvation energies of overall SSIPs used to represent the bead (eq 8).
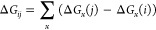
8

Here, we use SSIMPLE to calculate DPD repulsion parameters that describe the self-assembly of surfactant molecules in water at room temperature. However, SSIMPLE provides a general description of solvation of any molecule in any medium and can be applied to different temperatures.^35,36^ The approach may therefore also prove useful for the simulation of different kinds of supramolecular self-assembly processes that take place under quite different conditions.

**Figure 2:**
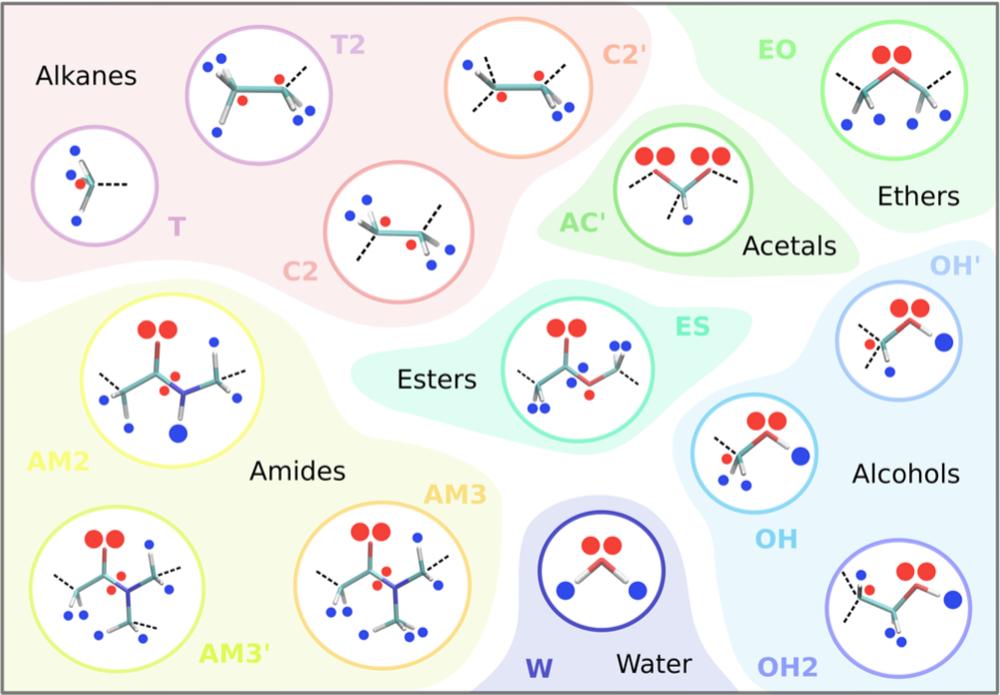
SSIP representation of the DPD beads used in this work. Red and blue dots show the negative and positive SSIPs, respectively. The larger value SSIPs highlighted in bold in Table 1 are shown as larger dots. The dotted lines indicate the connection with intramolecular beads.

Figure 2 illustrates the SSIP description used for each type of bead required to describe the molecules in Figure 1. The values of the SSIP interaction parameters ε were obtained using a footprinting algorithm applied to the molecular electrostatic potential surface (MEPS) of a closely related molecule calculated *ab initio* using density functional theory (B3LYP/631G*) on the 0.002 electron Bohr^–3^ electron density isosurface (Table 1, see the Supporting Information for details):^37^ methyl acetate for ES; *N*-methylacetamide for AM2; *N*,*N*-dimethylacetamide for AM3′ and AM3; methanol for OH1 and OH′; ethanol for OH2; methoxymethane for EO; dimethoxymethane for AC′; ethane for C2, C2′, and T2; and methane for T. Each SSIP has a footprint of 9 Å^2^ on the MEPS, so the total calculated surface area can be used to determine the number of SSIPs required to represent a molecule. The value of the MEPS at any location on the surface can be converted into an SSIP value using the quadratic relationships described previously.^37^ The footprinting algorithm optimizes the locations of the SSIPs on the MEPS in such a way that the net polarity of the SSIPs is maximized. To adjust the calculated SSIP description of these molecules to describe the relevant beads, the SSIPs positioned at the bond connection points between beads were removed. These missing interaction points are indicated with dotted bond lines in Figure 2.

**Table 1:** Van der Waals Volumes and SSIP Interaction Parameters for DPD Beads

bead	*v*_r_ (Å^3^)	positive SSIP ε_*i*_	negative SSIP ε_*i*_
W	42.0	**2.8, 2.8**	**–4.5, −4.5**
ES	68.8	0.4, 0.4, 0.4, 0.4, 0.2, 0.2	**–5.5, −5.5**, −2.6
EO	48.7	0.4, 0.4, 0.4, 0.4	**–5.3, −5.3**
AC′	31.9	0.4	**–4.4, −4.4, −4.4, −4.4**
AM2	73.2	**2.9**, 0.4, 0.4, 0.4, 0.4	**–7.9, −7.9**, −0.9, −0.9
AM3′	85.6	0.4, 0.4, 0.4, 0.4, 0.4, 0.4	**–7.9, −7.9**, −0.9, −0.9
AM3	92.1	0.4, 0.4, 0.4, 0.4, 0.4, 0.4, 0.4	**–7.9, −7.9**, −0.9, −0.9
T1	25.9	0.4, 0.4, 0.4	–0.3
T2	45.2	0.4, 0.4, 0.4, 0.4, 0.4	–0.3, −0.3
C2	38.9	0.4, 0.4, 0.4, 0.4	–0.3, −0.3
C2′	32.2	0.4, 0.4, 0.4	–0.3, −0.3
OH1	34.7	**2.7**, 0.4, 0.4	**–5.3, −5.3,** −0.3
OH′	28.1	**2.7**, 0.4	**–5.3, −5.3**, −0.3
OH2	43.8	**2.7**, 0.4, 0.4, 0.4	–**5**.**3**, –**5**.**3**, −0.3

a The distribution of SSIPs for each bead is illustrated in Figure 2 with SSIP values highlighted in bold represented by larger circles.

The concentration of the pure liquid of each bead is required for the calculation of transfer free energies. These values were estimated by using the concentration of a closely related molecule: methyl acetate for ES; *N*-methylacetamide for AM2, *N*,*N*-dimethylacetamide for AM3′ and AM3; methanol for OH1 and OH′; ethanol for OH2; half of the concentration of dimethoxyethane for EO and AC′; one-quarter of the concentration of *n*-octane for C2, C2′, and T2; and one-eighth of the concentration of *n*-octane for T.

The van der Waals volume of each bead is required for the calculation of the repulsion parameters using eq 5, and these values were estimated based on the volume of the 0.002 electron Bohr^–3^ electron density isosurface of closely related molecules calculated using the density functional theory (B3LYP/631G*). For terminal beads with a single connection point, the volume was obtained from half the volume of the fragment dimer. T was obtained from half the volume of ethane, T2 from half the volume of *n*-butane, and OH from half the volume of 1,2-ethandiol. For beads such as C2 and EO, which have two connection points, volumes were obtained from the homologous series of alkanes and ethylene glycols, respectively. The difference between the volume of C2 and the volume of ethane (6.5 Å^3^) is the volume correction required to adjust the volume of a molecule terminated with two methyl groups to a bead with two connection points. This approach was used to calculate the volumes of the ES, AM2, AM3, and AM3′ beads from the volumes of methyl acetate, *N*-methyl acetamide, and *N*,*N*-dimethyl acetamide. The volume for OH′ was obtained from one-sixth of the volume of myo-inositol. The bead volumes are reported in Table 1.

The bead radii *R*_*ij*_ and the repulsion parameters *a*_*ij*_ and Δ*a*_*ij*_ calculated for all bead combinations are reported in Tables 2, 3, and 4. The repulsion parameter values provide some insights into the expected behavior of the surfactants in aqueous solutions. For example, if we compare the three different amide beads, the two tertiary amides make more favorable interactions with the water bead W (Δ*a*_AM3′–W_ = −11.64 *k*_B_*T* and Δ*a*_AM3–W_ = −10.30 *k*_B_*T*) than the secondary amide (Δ*a*_AM2–W_ = −7.93 *k*_B_*T*). This result might seem counterintuitive because the NH group in the secondary amide is a good H-bond donor, which should promote the interaction with water. However, there are stronger amide–amide H bonds in the pure liquid of the secondary amide, which leads to a less favorable change in free energy for transfer into water. The transfer of tertiary amides into water is more favorable because there is no loss of amide–amide H bonds, only a gain of H-bonding interactions with the water H-bond donors.

**Table 2:** *R*_*ij*_ Values for All Bead Combinations

	W	OH1	OH′	OH2	ES	EO	AC′	C2	C2′	T1	T2	AM2	AM3′	AM3
W	1.000													
OH1	0.990	0.980												
OH′	0.975	0.965	0.949											
OH2	1.003	0.996	0.981	1.012										
ES	1.071	1.061	1.045	1.077	1.141									
EO	1.058	1.048	1.033	1.064	1.129	1.116								
AC′	0.976	0.966	0.951	0.982	1.047	1.034	0.952							
C2	1.037	1.027	1.012	1.043	1.108	1.095	1.013	1.074						
C2′	0.998	0.988	0.972	1.004	1.068	1.056	0.974	1.035	0.995					
T1	0.978	0.968	0.952	0.984	1.048	1.036	0.954	1.015	0.975	0.955				
T2	1.049	1.039	1.024	1.055	1.120	1.107	1.025	1.086	1.047	1.027	1.098			
AM2	1..086	1.076	1.061	1.092	1.157	1.144	1.062	1.123	1.084	1.064	1.135	1.172		
AM3′	1.118	1.108	1.093	1.124	1.189	1.176	1.094	1.155	1.116	1.096	1.167	1.204	1.236	
AM3	1.133	1.123	1.108	1.139	1.204	1.191	1.109	1.170	1.131	1.111	1.182	1.219	1.251	1.266

**Table 3:** *a*_*ij*_ Values for All Bead Combinations

	W	OH1	OH′	OH2	ES	EO	AC′	C2	C2′	T1	T2	AM2	AM3′	AM3
W	25.00													
OH1	18.17	14.00												
OH′	15.09	13.86	14.00											
OH2	22.20	16.24	15.95	18.00										
ES	22.53	18.43	19.56	19.63	22.00									
EO	21.81	18.17	19.92	20.31	24.01	22.50								
AC′	7.74	17.42	19.42	17.42	22.69	24.37	22.50							
C2	45.45	27.13	28.77	27.09	21.50	23.78	18.17	22.00						
C2′	45.50	29.76	29.37	27.38	22.26	24.49	19.45	21.95	22.00					
T1	46.35	27.49	28.85	27.59	21.61	24.18	20.38	22.92	20.89	24.00				
T2	45.44	26.79	28.28	27.59	21.67	24.46	17.32	21.97	21.13	23.76	24.00			
AM2	15.57	14.63	14.25	16.84	25.39	26.86	26.23	28.75	32.32	29.11	28.41	22.00		
AM3′	11.86	11.03	10.62	13.20	22.05	23.94	22.87	21.63	21.92	22.00	21.28	21.77	22.00	
AM3	13.20	11.52	11.00	13.71	21.97	23.84	22.51	21.83	22.17	22.32	21.56	21.89	21.98	22.00

**Table 4:** Δ*a*_*ij*_ Values for All Bead Combinations

	W	OH1	OH′	OH2	ES	EO	AC′	C2	C2′	T1	T2	AM2	AM3′	AM3
W	0.00													
OH1	–1.33	0.00												
OH′	–4.41	–0.14	0.00											
OH2	0.70	0.24	–0.05	0.00										
ES	–0.97	0.43	1.56	–0.37	0.00									
EO	–1.94	–0.08	1.67	0.06	1.76	0.00								
AC′	–16.01	–0.83	1.17	–2.83	0.44	1.87	0.00							
C2	21.95	9.13	10.77	7.09	–0.50	1.53	–4.08	0.00						
C2′	22.00	11.76	11.37	7.38	0.26	2.24	–2.80	–0.05	0.00					
T1	21.85	8.49	9.85	6.59	–1.39	0.93	–2.87	–0.08	–2.11	0.00				
T2	20.94	7.79	9.28	6.59	–1.33	1.21	–5.93	–1.03	–1.87	–0.24	0.00			
AM2	–7.93	–3.37	–3.75	–3.16	3.39	4.61	3.98	6.75	10.32	6.11	5.41	0.00		
AM3′	–11.64	–6.97	–7.38	–6.80	0.05	1.69	0.62	–0.39	–0.08	–1.00	–1.72	–0.23	0.00	
AM3	–10.30	–6.48	–7.00	–6.29	–0.03	1.59	0.26	–0.17	0.17	0.68	–1.44	–0.11	–0.02	0.00

### Bond Parameters

In a DPD simulation, the interaction between two bonded beads includes both the harmonic spring potential and the nonbonded repulsion. Thus, the value of *r*_0_ that should be used in the bond length potential given by eq 2 is not the same as the bond length observed experimentally. For the equilibrium bond length obtained in a DPD simulation to match the experimental bond length, the value of *r*_0_ must be slightly shorter than the desired value of *r*_*ij*_, which we will define as *r*_target_. For beads where atoms are linearly connected, the empirical approach introduced by Anderson *et al.,* where the bond length parameter *r*_0_ depends on the number of heavy atoms, has been shown to work reasonably well. However, for beads representing more complex fragments, such as amides and esters, the bond distance does not grow linearly with the number of heavy atoms, so a different approach is required. We propose an improved method which matches the potential energy minimum in the interaction between bonded DPD beads with a target bond length computed atomistically. The relationship between the potential energy and bond length can be written combining eqs 1 and 2 as 9.
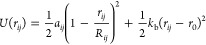
9

Setting the first derivative of eq 9 to zero gives the value of *r*_0_ required to obtain any desired bond length *r*_target_ (eq 11).
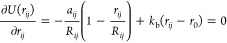
10
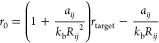
11

**Figure 3:**
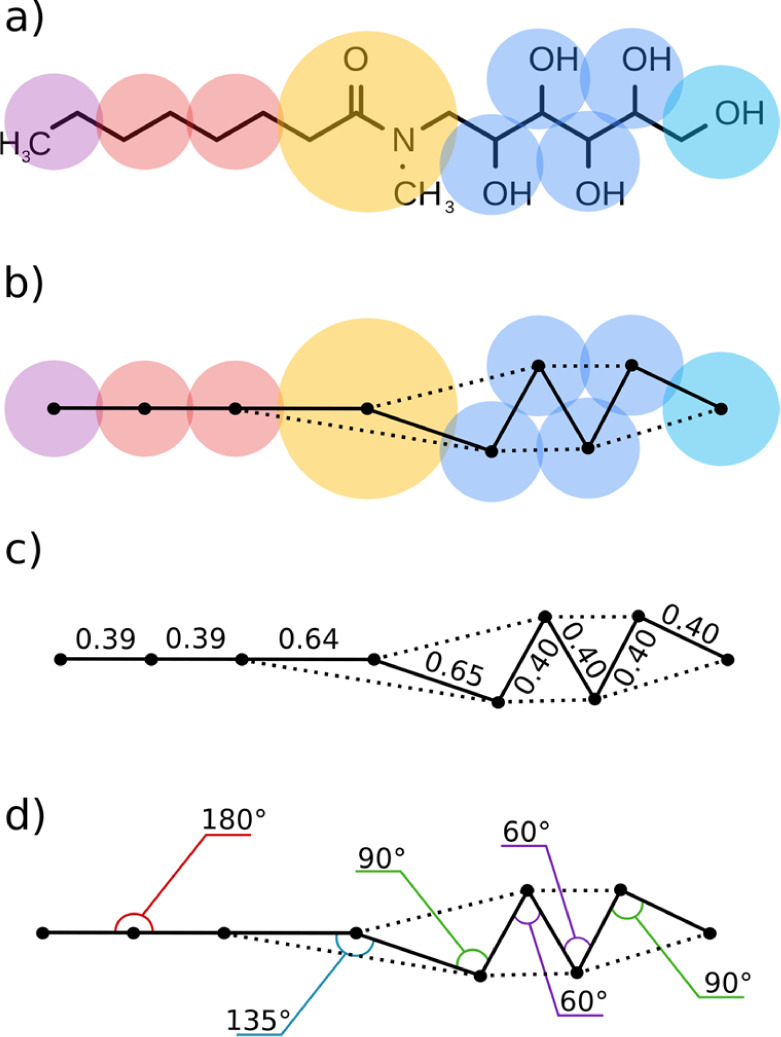
DPD parameters for MEGA8: (a) chemical structure and (b) the CG description with bead color coded according to Figure 1. The full lines indicate where bond distance parameters are required and the dashed lines represent where bond angle parameters are required. (c) Bond length parameters *r*_0_. (d) Bond angle parameters θ_0_.

The values of *R*_*ij*_ are listed in Table 2, and the values of *r*_target_ were calculated using molecular mechanics models. For each pair of covalently bonded beads in each of the surfactant structures, the 3D atomic structure of the molecule was built and optimized using the MMFF94 force field. The target distances between all bead pairs were obtained using the centers of mass of the corresponding fragments as illustrated for MEGA8 in Figure 3, and eq 11 was used to calculate the corresponding values of *r*_0_. For very flexible fragments, such as the polyol chains present in the XYL, MEGA, and HEGA systems, molecular mechanics calculations using the full surfactant structure are less useful because a range of different parameters are obtained depending on which beads are selected. The required OH′–OH′ bond parameter was therefore calculated using ethylene glycol. The values of *r*_target_ and *r*_0_ are reported in Table 5. The bond angle parameters θ_0_ were calculated directly from the molecular structures as illustrated in Figure 3 (see the Supporting Information for details). For amides, where different conformers are possible, parameters were calculated for both the *cis* and the *trans* conformations. However, the results of DPD simulations carried out on these two different representations were very similar for all three MEGA systems. The calculated values of CMC and *N*_agg_ do not appear to be very sensitive to the amide conformation (see Supporting Information, Table S2). Given that the population of *cis* and *trans* conformers is also a variable that is difficult to determine, only the most extended *trans* conformation was used for all of the amides in the DPD simulations described below.

**Table 5:** Bond Parameters *r*_target_ and *r*_0_

surfactant	bead *i*	bead *j*	*r*_target_	*r*_0_
MEA	AM2	C2	0.65	0.58
MEA	AM2	OH	0.68	0.65
TEDA	AM2	EO	0.78	0.73
DEA	AM3′	C2	0.67	0.62
DEA	AM3′	OH	0.67	0.64
HEGA	AM3′	OH	0.67	0.64
MEGA	AM3	C2	0.68	0.64
MEGA	AM3	OH′	0.68	0.65
XYL	ES	C2	0.60	0.55
XYL	ES	OH′	0.65	0.60
XYL	OH′	OH′	0.45	0.40

### DPD Simulations

DPD simulations were performed on all of the surfactant systems shown in Figure 1 for hydrocarbon chain lengths from C8 to C12. Each solvent bead represented two molecules of water, with the value of *R*_*ij*_ set equal to *r*_c_, the DPD length unit. The dimensionless bead density was set to ρ*r*_c_^3^ = 3 with ρ being the bead density. This can be translated in common units as *r*_c_ = 5.64 Å.^27^ Simulations were run in a cubic box of size 40*r*_c_ with a total of 192,000 beads. Simulations were run for 4 × 10^6^ timesteps with a timestep equal to 0.01 in DPD units. The DL_MESO package (version 2.7)^38^ was used to perform all the simulations, and the UMMAP tool^39^ was used in combination with purpose written scripts for the analysis. The trajectory files were collected every 1000 timesteps. The standard velocity Verlet integration was used.^40^ Simulations were run at 4, 5, and 6 wt % to obtain the CMC values. For investigating the *N*_agg_ and micelle structure, concentrations were matched with those reported experimentally (see the Supporting Information). The simulations were evaluated by comparison with three experimental properties: the CMC, the mean aggregation number (*N*_agg_), and the aggregate shape.

### Critical Micelle Concentration

The CMC value was obtained from averaging the concentration of free surfactants plus submicellar micelles for each step of the DPD simulation after equilibrium had been reached. For all systems, a stable value of free surfactants was reached between 2.0 × 10^5^ and 4.0 × 10^5^ timesteps. CMC values were collected after 5.0 × 10^5^ timesteps for all systems. As previously reported,^14^ the value of *N*_cut_ used to discriminate between premicelles and stable micelles was obtained from the aggregation number distribution *P*(*N*). For highly soluble surfactants, *N*_cut_ is identified as a local minimum in the *P*(*N*) distribution, while there is usually a clear gap between the two populations for less soluble surfactants. The CMC values for very soluble surfactants such as MEGA8 and HEGA8 had shown some dependency on the value of *N*_cut_ due to the overlap of premicelle and stable micelle populations in the *P*(*N*) distribution.^17^ Examples are reported in the Supporting Information.

**Figure 4:**
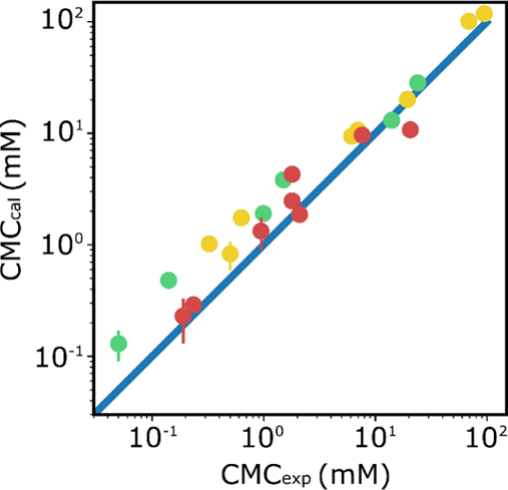
Comparison of calculated and experimental CMC values. In red, ester-linked surfactants, in yellow, amide-linked surfactants, and in green, sugar-linked surfactants.

CMC values for each surfactant are reported in Table 6, and Figure 4 shows the relationship between the calculated CMC values and the available experimental data. There is a good correlation. The main factor which determines the CMC is the length of the hydrophobic chain, and in accordance with the Stauff–Klevens rule, the CMC drops by about an order of magnitude for every two CH2 groups added. Differences in the shape of the tail do not significantly change the CMC value, and the CYGLU and CYMAL systems, which have a terminal cyclohexyl group, have comparable CMC values to related surfactants which have a linear alkyl chain with the same number of carbons. There is some effect of the nature of the hydrophilic head group on the CMC values, and the amide surfactants show consistently higher solubility in water than the sugar and ester surfactants. This result can be directly related to the SSIP description of the beads used in the DPD simulations. All of the amide beads show a high affinity for water due to the favorable interaction between the two most negative SSIPs of the carboxamide group (ε_*i*_ = −7.9) and the positive SSIPs of the aqueous solvent (ε_*i*_ = +2.8). The CMC values for the longer chain length sugar and amide surfactants (green and yellow data points in Figure 4) tend to be overestimated, which may be related to the fact that some of these systems do not fully equilibrate on the time scale of the DPD simulations (see below).

**Table 6:** Calculated and Experimental Values of CMC and *N*_agg_

Surfactant	CMC calc. (mM)	CMC exp. (mM)	*N*_agg_ calc.	range of *N*_agg_ calc.	*N*_agg_ exp.
GLY8	13.06 ± 0.67	14^41^	70	56–84	
GLY10	1.92 ± 0.07	0.99^41^	878	361–1636	
GLY12	0.13 ± 0.04	0.053^41^	659	318–1439	
XYL8	28.41 ± 3.42	24^41^	29	24–33	
XYL10	3.81 ± 0.31	1.5^41^	47	42–49	
XYL12	0.48 ± 0.08	0.14^41^	75	69–76	
MEA8	63.12 ± 1.81		37	33–44	
MEA10	6.35 ± 0.64		155	105–210	
MEA12	0.96 ± 0.26		992	455–1646	
DEA8	71.50 ± 2.4		23	20–26	
DEA1O	9.28 ± 0.22		45	39–51	
DEA12	1.33 ± 0.44	0.07,^42^ 1.83^43^	98	75–124	
MEGA8	101.03 ± 3.56	51.5,^44^ 70,^45^ 74.2,^46^ 79^47^	15	9–18	24,^48^ 85^49^
MEGA10	9.44 ± 0.45	4.82,^44^ 6.8,^46^ 6–7^47^	26	26–28	28,^50^ 75^48^
MEGA12	1.02 ± 0.11	0.35^51^	35	26–36	
HEGA8	118.6 ± 8.2	80, 109^47^	18	16–18	
HEGA10	10.65 ± 1.51	7^47^	23	19–24	
HEGA12	1.75 ± 0.19	0.63^52^	29	27–33	
TEDA12	0.83 ± 0.24	0.5^53^	33	32–34	130 ± 10^53^
GLUCO8	10.74 ± 0.25	20–25,^54^18–26^47^	38	36–40	27–100^47^
GLUCO10	1.87 ± 0.09	2,^54^ 2.2^47^	70	63–74	
GLUCO12	0.23 ± 0.11	0.19^47^	252	139–435	200–400^55^
MALTO8	20.1 ± 0.17	19.5^47^	19	18–20	35–47,^47^ 26^56^
MALTO10	2.48 ± 0.23	1.8^47^	27	26–29	69,^47^ 82–103^56^
MALTO12	0.29 ± 0.03	0.17,^47^ 0.3^53^	33	32–38	78–149,^47^75–130^53^
CYGLU4	4.3 ± 0.38	1.8^47^	43	38–46	
CYMAL4	9.64 ± 0.6	7.6^47^	27	23–28	25–45^47^

### Aggregation Number

The mean aggregation number is defined as the weighted average number of molecules per micelle, and values were calculated from the simulations using eq 12.
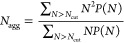
12

The value of *N*_agg_ equilibrated much more slowly than the value of CMC in the DPD simulations, requiring from 1.0 × 10^6^ to 3.5 × 10^6^ timesteps. Due to the dynamic breaking and forming of micelles, the value of *N*_agg_ tends to fluctuate even after convergence, so we report the range of *N*_agg_ values observed in each step after equilibrium was reached, as well as the average value (Table 6). For MALTO12 and TEDA12, convergence was never completely reached. The exchange rate of molecules between micelles depends directly on monomer solubility and the number of micelles present in the simulation, both of which decrease with the length of the hydrophobic tail. This effect leads to an underestimate value of *N*_agg_ in DPD simulations of less soluble surfactants.^17,27,57^

**Figure 5:**
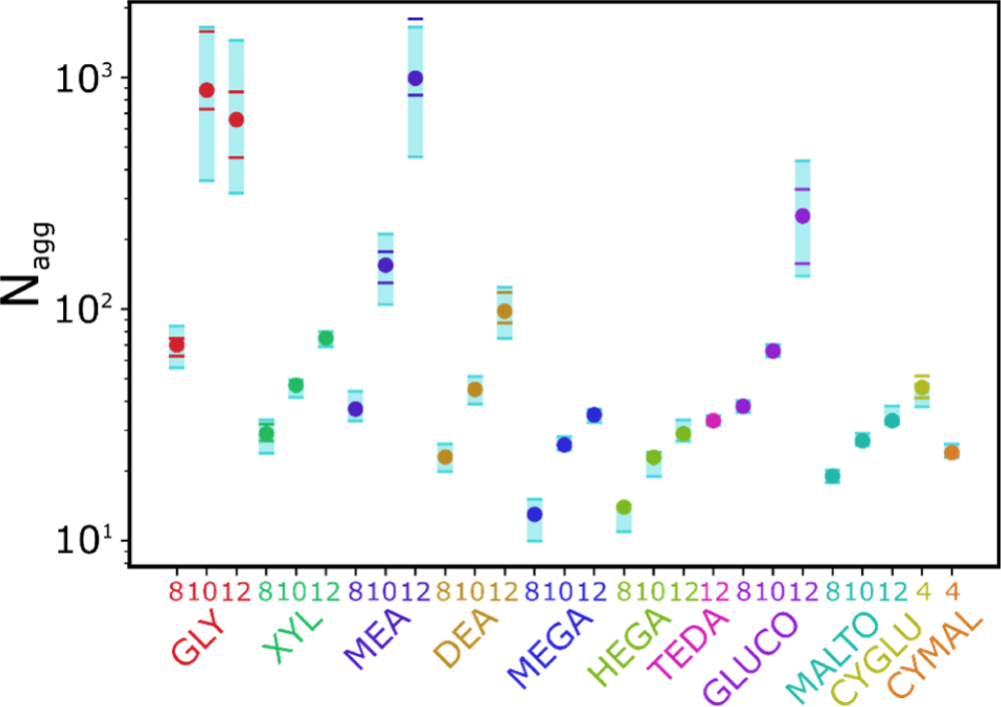
Calculated aggregation number for simulations at 5 wt % surfactant. Dots represent the average value of *N*_agg_ obtained after equilibration, the bars show the standard deviation (omitted if smaller than the dot size), and the range of *N*_agg_ values is shaded light blue.

The aggregation numbers obtained from the DPD simulations are illustrated in Figure 5. Within a surfactant family, there is a clear increase in the value *N*_agg_ with the length of the hydrophobic tail. However, the magnitude of the effect is strongly dependent on the nature of the hydrophilic head group. For example, the MEA systems show a very steep dependence of *N*_agg_ on the hydrocarbon tail length, with an increase of nearly an order of magnitude for each CH2 group added, whereas for the XYL systems, *N*_agg_ does not even double for each added CH2 group. The fact that GLY10 and GLY12 give very similar values of *N*_agg_, which are an order of magnitude larger than the value for GLY8, suggests that the value for GLY12 is significantly underestimated. However, the computational expense of simulating much larger aggregates would be excessive. The largest aggregates (*N*_agg_ ≈ 1000) are formed by surfactants that have long hydrophobic tails and small hydrophilic head groups, GLY10, GLY12, and MEA12. These very large values of *N*_agg_ are indicative of potentially unlimited growth and the formation of worm-like micelles in the case of rods or vesicles/lamellar phase in the case of discs. Conversely, surfactants with a short hydrophobic tail and a large hydrophilic head group, MEGA8 and HEGA8, form the smallest aggregates with values of *N*_agg_ less than 20.

The calculated values are compared with the corresponding experimental data, where available, in Table 6. The experimental values often span a wide range due to the differences between the techniques that have been used to measure *N*_agg_. For example, the value of *N*_agg_ reported for MEGA8 from DLS measurements is 24,^48^ while 85 was obtained from spectrofluorimetry.^49^ Similarly, for MEGA10, the value measured by isothermal titration calorimetry was 28,^50^ compared with 75 from DLS.^48^ In general, there is a reasonable agreement between the experimental range and the calculated range of values for *N*_agg_, with the exception of TEDA12 and MALTO12, where the simulation failed to equilibrate.

### Aggregate Shape

**Figure 6:**
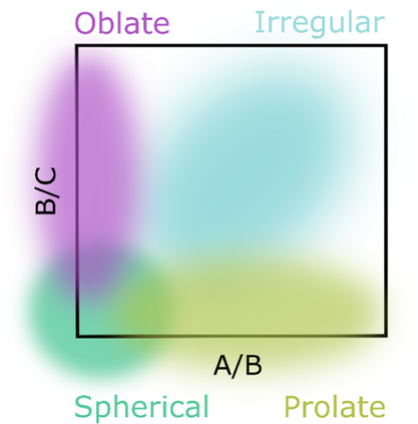
Supramolecular structures formed by surfactants. *A*, *B*, and *C* are spheroid semi-axes. The region where spherical micelles are located is highlighted in green, in yellow prolate or rod-like structures, in purple oblate or disc-like structures, and in light blue irregular ellipsoids.

Surfactants can form a large variety of different aggregates in aqueous solutions, and the shape of the aggregate can often change as a function of concentration. For example, a micelle- to rod-like transition has been reported for many systems.^58−61^ Mixtures of different aggregate structures can coexist, which leads to ambiguities in the assignment of aggregate shape whether using experimental or computational methods. In this work, we approach the problem in the following manner. For each timestep after equilibration, the semi-axes *A*, *B*, and *C* used to describe a spheroid are collected for each aggregate (*N* > *N*_cut_), where *A* is the biggest axis and *C* is the smallest axis. The ratios *A*/*B* and *B*/*C* are plotted as a 2D histogram, as illustrated in Figure 6. There are three limiting situations: *A* = *B* = *C* describes a sphere, that is, a micelle (green region in Figure 6); *A* > *B* = *C* describes a prolate shape, that is, a rod-like structure (yellow region in Figure 6); and *A* = *B* > *C* represents an oblate structure, that is, a disc-like structure (purple region in Figure 6). The area where *A* > *B* > *C* in Figure 6 (blue) is populated by a range of different structures that vary from simple irregular spheroids to complex branched rod-like structures.

**Figure 7:**
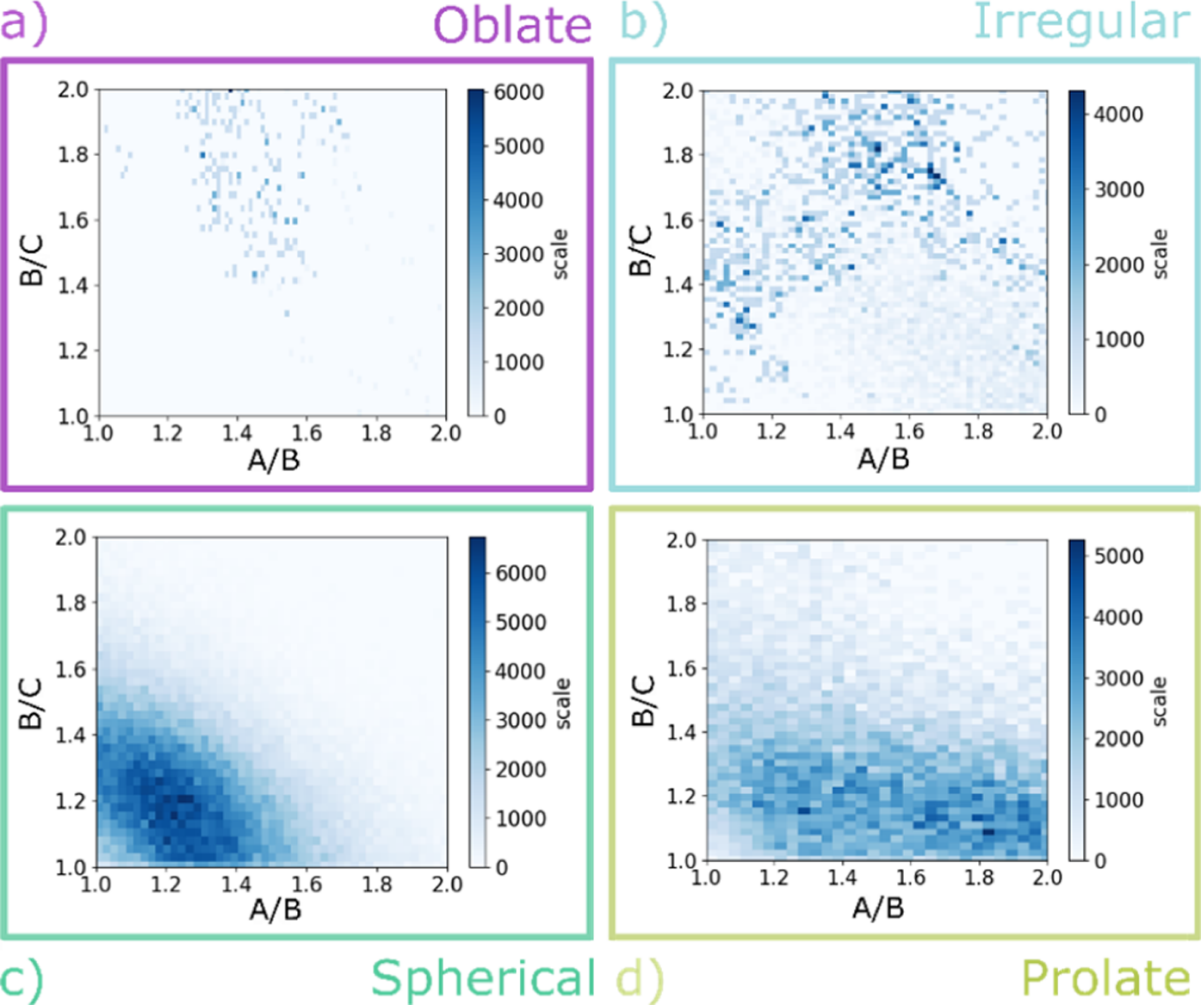
Effect of the head group on the shape of the supramolecular aggregate for four different C12 surfactants at 5 wt %. 2D histograms showing the populations of different shaped aggregates defined using the spheroid semi-axes *A*, *B*, and *C* for (a) GLY12, (b) MEA12, (c) MALTO12, and (d) DEA12.

**Figure 8:**
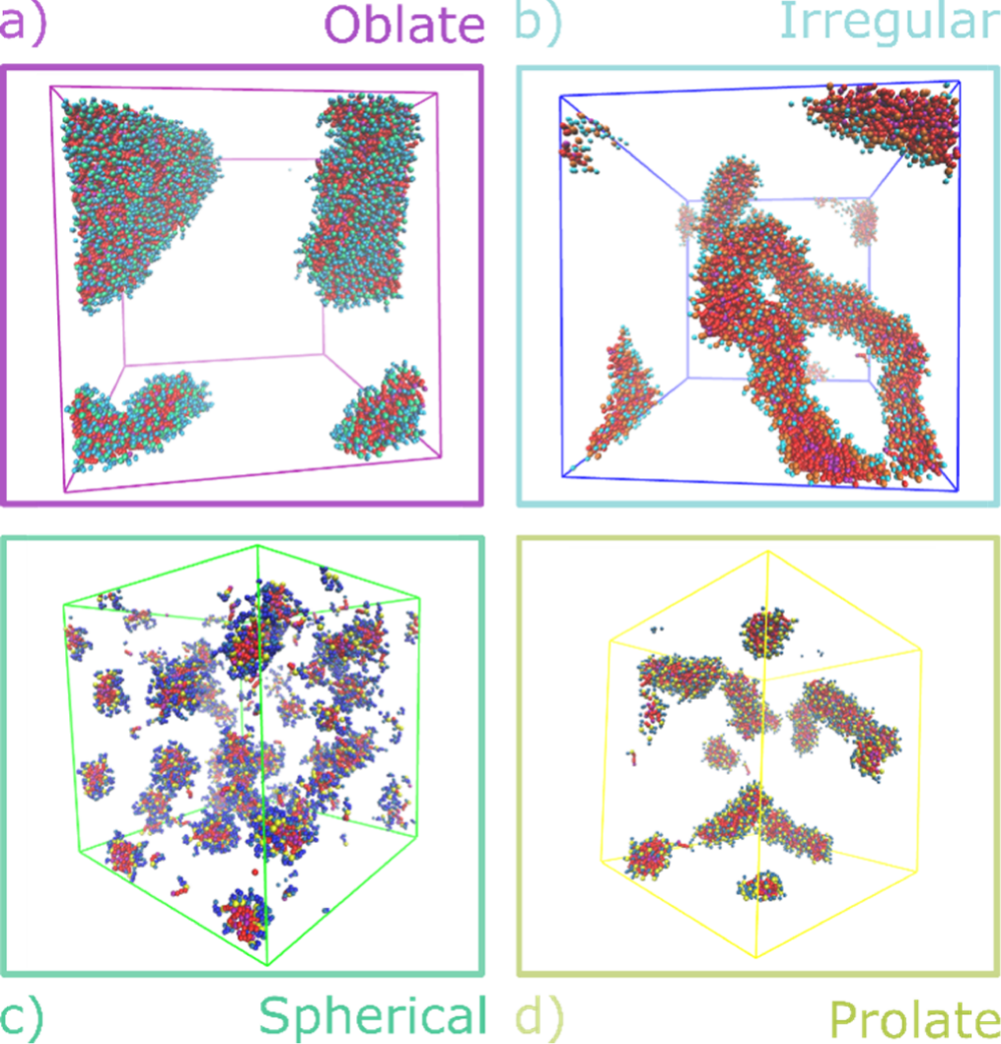
Snapshots of DPD simulations for four different C12 surfactants at 5 wt % concentration after equilibration. (a) GLY12 forms fragments of bilayers, (b) MEA12 forms worm-like structures, (c) MALTO12 forms micelles, and (d) DEA12 forms rod-like structures.

Figures 7 and 8 show how the nature of the head group affects the shape of the aggregate formed for four different C12 surfactants (results for all of the surfactants are reported in the Supporting Information). The GLY surfactants have the smallest head group, and Figures 7a and 8a show that this leads to a bilayer structure for GLY12 (visible as a fragment in Figure 8a).^41^ In contrast, the MALTO surfactants have a large hydrophilic head group, and all form spherical micelles (Figures 7c and 8c). Similarly, the DPD simulations indicate that the MEGA and HEGA systems form small spherical micelles in agreement with experimental reports based on measurements of the hydrodynamic radii of aggregates.^62,63^ Surfactants with smaller head groups, such as MEA and DEA, form aggregates that show a stronger dependency on the hydrophobic tail length. The DEA systems go from small spherical micelles for DEA8 to rod-like aggregates for DEA12 (Figures 7d and 8d). The effect is even more pronounced in the MEA systems, where there is a transition from spherical micelles for MEA8 to worm-like structures for MEA12 (Figures 7b and 8b). The GLUCO systems also show a range of different structures, consistent with SAXS measurements, which identified the presence of elongated micelles with an oblate form.^56^ The other two surfactants CYGLU-4 and CYMAL-4 generally form spherical micelles.

## Conclusions

The methods described here provide a general approach to determining DPD parameters for neutral organic compounds of arbitrary structure. The parameters required to describe the bonded interactions between DPD beads were calculated from molecular mechanics structures of the surfactant molecules. The parameters required to describe the nonbonded interactions were calculated from SSIP descriptions of molecular fragments that represent individual beads. The SSIPs were obtained from molecular electrostatic potential surfaces calculated using density functional theory and used in the SSIMPLE algorithm to calculate transfer free energies between different bead liquids. This approach was used to calculate DPD parameters for a range of different types of surfactants, which include ester, amide, and sugar moieties. The parameters were used to simulate the self-assembly properties in aqueous solutions, and comparison of the results for 27 surfactants with the available experimental data shows that these DPD simulations accurately predict CMCs, aggregation numbers, and the shapes of the supramolecular assemblies formed. The methods for calculation of DPD parameters directly from the chemical structure offer a general solution to obtaining the parameters required for simulation of uncharged organic molecules.

## Supporting Information Available

Details of calculation of the fragment volumes, determination of *N*_cut_, 2D histograms of assembly shape distribution, DPD parameters for bonded interactions, and computational details (PDF)
