# Supplementary material for: Translation of Chemical Structure into Dissipative Particle Dynamics Parameters for Simulation of Surfactant Self-Assembly
Source: J Phys Chem B. 2021 Apr 13;125(15):3942–52. doi: 10.1021/acs.jpcb.1c00480 (PMC8154614; doi:10.1021/acs.jpcb.1c00480)
Supplement: Supplementary file 1 — jp1c00480_si_001.pdf [file jp1c00480_si_001.pdf]

Translation of Chemical Structure into Dissipative Particle Dynamics Parameters For Simulation of Surfactant Self-Assembly.

Ennio Lavagnini,[a] Joanne L. Cook,[b] Patrick B. Warren,[b,c] and Christopher A. Hunter [a*]

[a] Department of Chemistry, University of Cambridge, Lensfield Road, Cambridge CB2 1EW, United Kingdom.
[b] Unilever R&D Port Sunlight, Quarry Road East, Bebington CH63 3JW, United Kingdom.
[c] The Hartree Centre, STFC Daresbury Laboratory, Warrington WA4 4AD, United Kingdom.

Supplementary Information

Density Functional Theory calculations **S2**
Determination of N_{cut} **S3**
2D histograms of assembly shape distribution **S4**
Parameters for bonded interactions **S13**
Amide conformers **S26**

Density Functional Theory Calculations

DFT calculations were carried out with Gaussian9-D01 with B3LYP/631G*. The molecules were constructed using molecular mechanics, the structures were optimised using DFT, and the MEPS was calculated on the 0.002 electron Bohr^{-3} electron density isosurface.

Footprinting of the DFT MEPS was carried out using the method described in Calero, C. S.; Farwer, J.; Gardiner, E. J.; Hunter, C. A.; Mackey, M.; Scuderi, S.; Thompson, S.; Vinter, J.G. Footprinting Molecular Electrostatic Potential Surfaces for Calculation of Solvation Energies. *Phys. Chem. Chem. Phys,* **2013** *15* (41), 18262. https://doi.org/10.1039/c3cp53158a (internal git version 44285baa). The resulting SSIP distribution for each molecule is shown in Figure S3 and the values of the SSIPs are given in Table 1 of the main text.

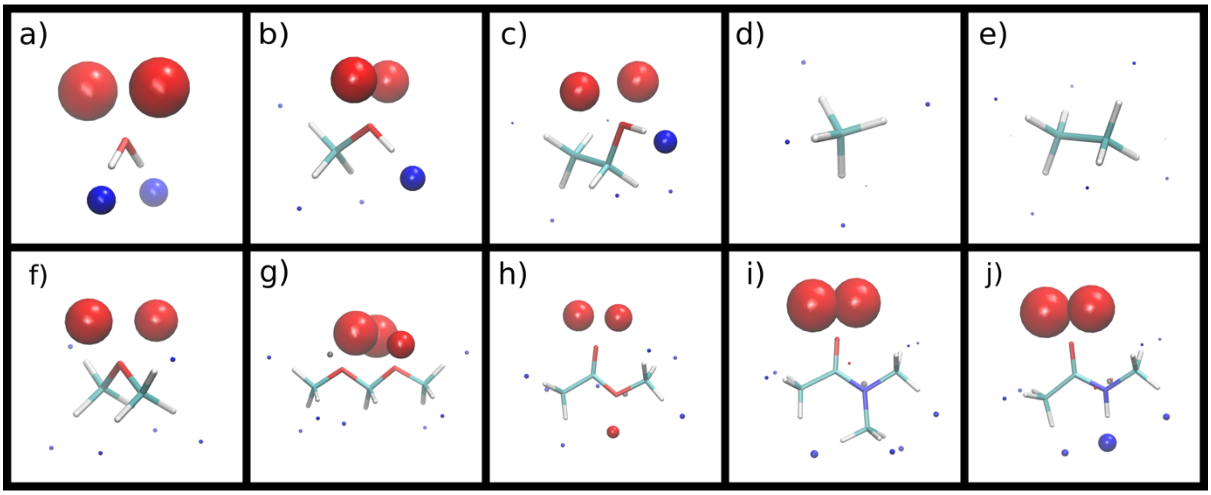

Figure S1: SSIPs calculated by footprinting the electrostatic potential calculated on 0.002 Bohr Å^{-3} electron density isosurfaces calculated using DFT and B3LYP/631G. (a) water (b) methanol (c) ethanol (d) methane (e) ethane (f) methoxymethane (g) dimethoxymethane (h) methyl acetate (i) N,N-dimethyl acetamide (j) N-methyl acetamide.*

Solvation energies were calculated using the SSIMPLE method described in Hunter, C. A. A Surface Site Interaction Model for the Properties of Liquids at Equilibrium. *Chem. Sci.* **2013**, *4* (4), 1687. https://doi.org/10.1039/c3sc22124e (internal git version 9ae19fda).

DPD simulations were run on a GNU/Linux centos 6.10 system powered by two eight-core Intel Xeon E5-2650 v2 (2.60GHz, Ivy Bridge) CPUs with 128GB of RAM.

Determination of N_{cut}

(a)

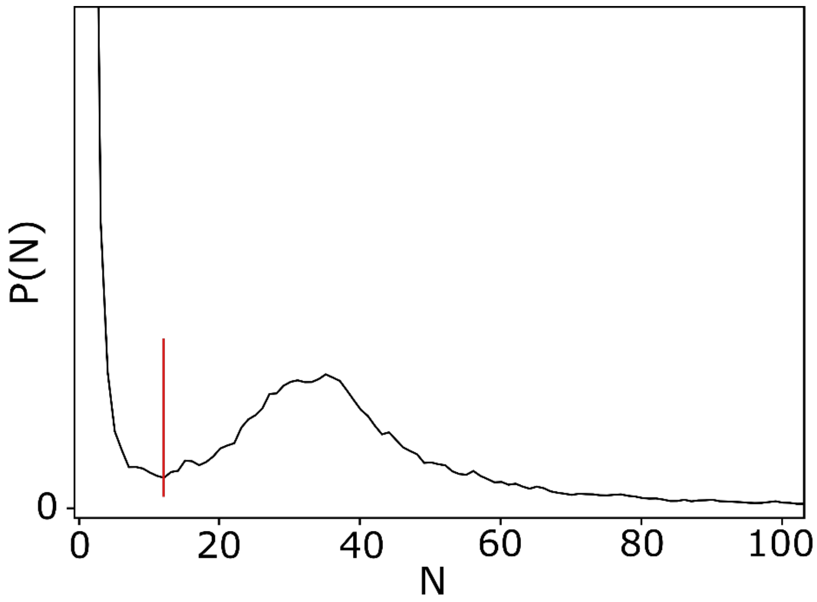

(b)

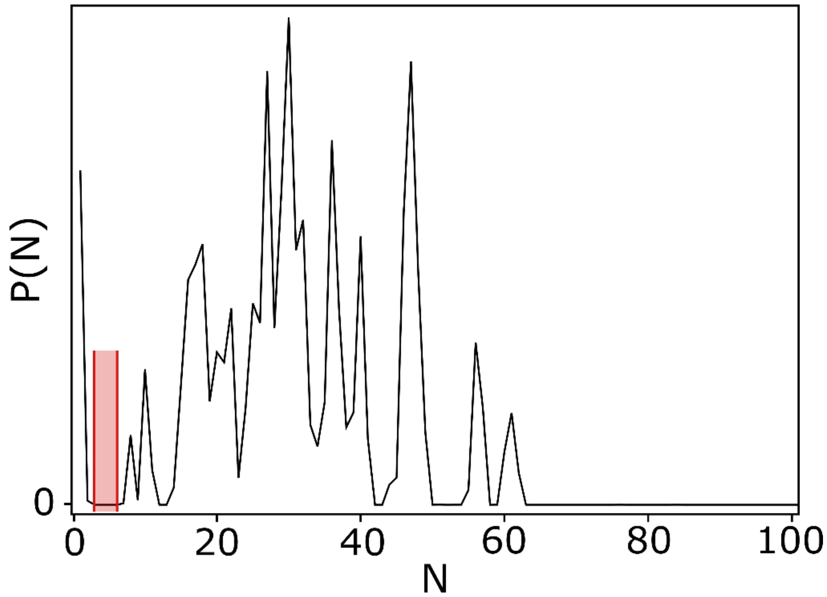

Figure S1: (a) Aggregation distribution (N) for MEA8 surfactant at 5%wt. Ncut is highlighted in red. (b) Aggregation distribution (N) for MALTO12 surfactant at 5%wt. Ncut gap is highlighted in red. As we have shown previously, this kind of jagged P(N) distribution does not significantly affect the accuracy of the values of CMC and N_{agg} *that are calculated from it (publication (see J. Phys. Chem. B 2020, 124, 5047)).*

2D histograms of assembly shape distribution

GLY histograms

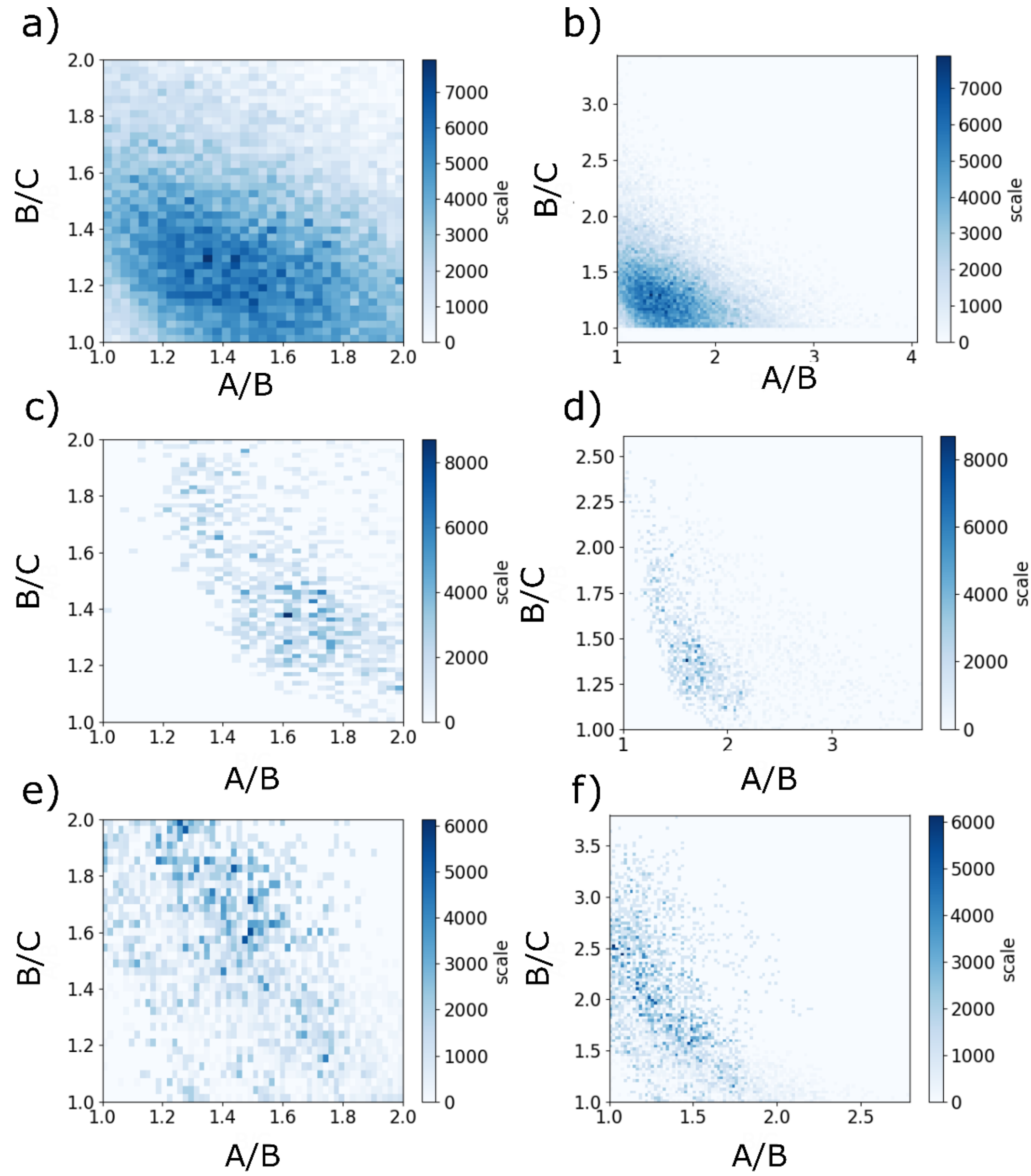

Figure S2: Histograms for GLY systems. a-b) GLY8 histograms. c-d) GLY10 histograms. e-f) GLY12 histograms.

XYL histograms

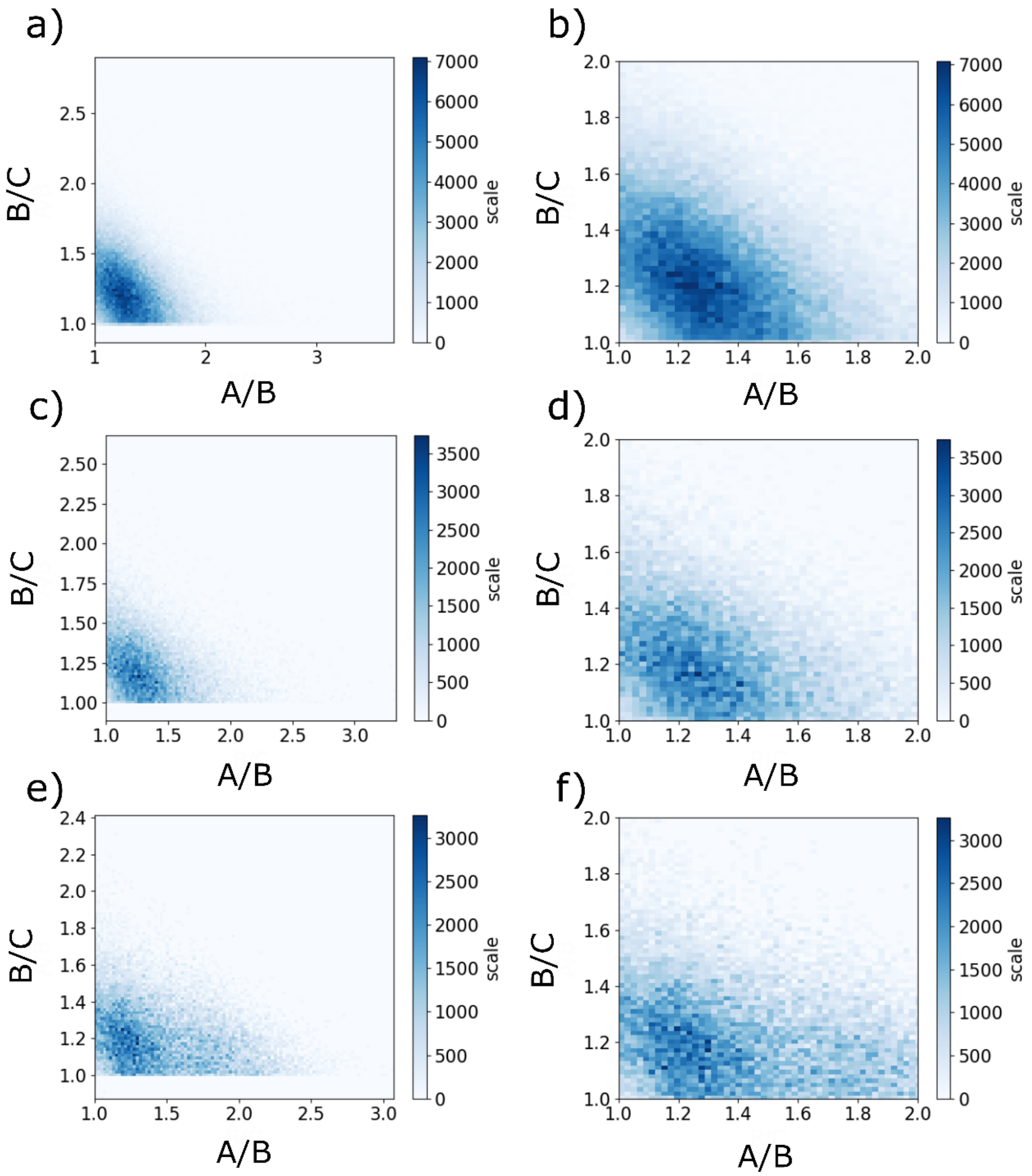

Figure S3: Histograms for XYL systems. a-b) XYL8 histograms. c-d) GLY10 histograms. e-f) GLY12 histograms.

MEA histograms

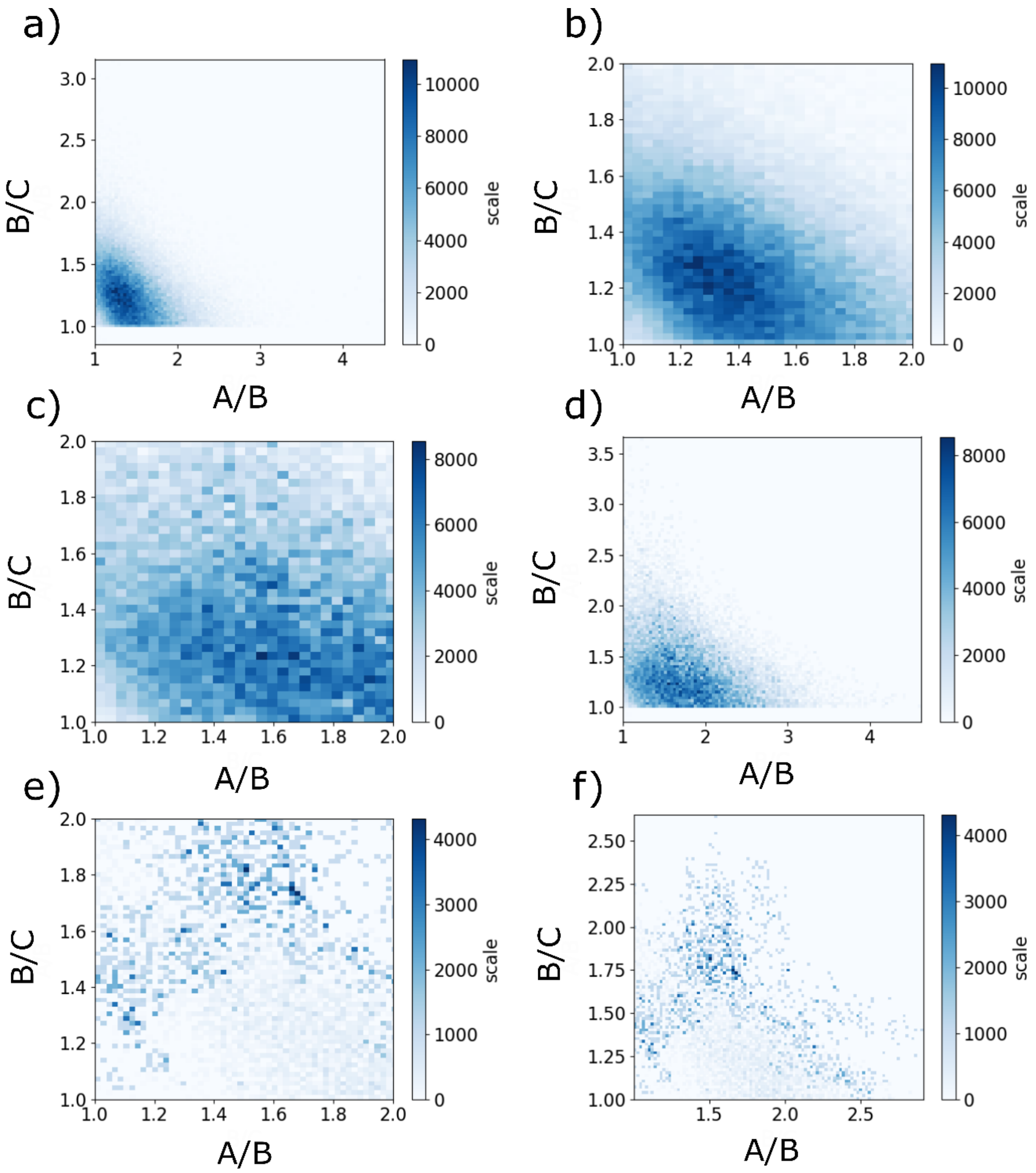

Figure S4: Histograms for MEA systems. a-b) MEA8 histograms. c-d) MEA10 histograms. e-f) MEA12 histograms.

DEA histograms

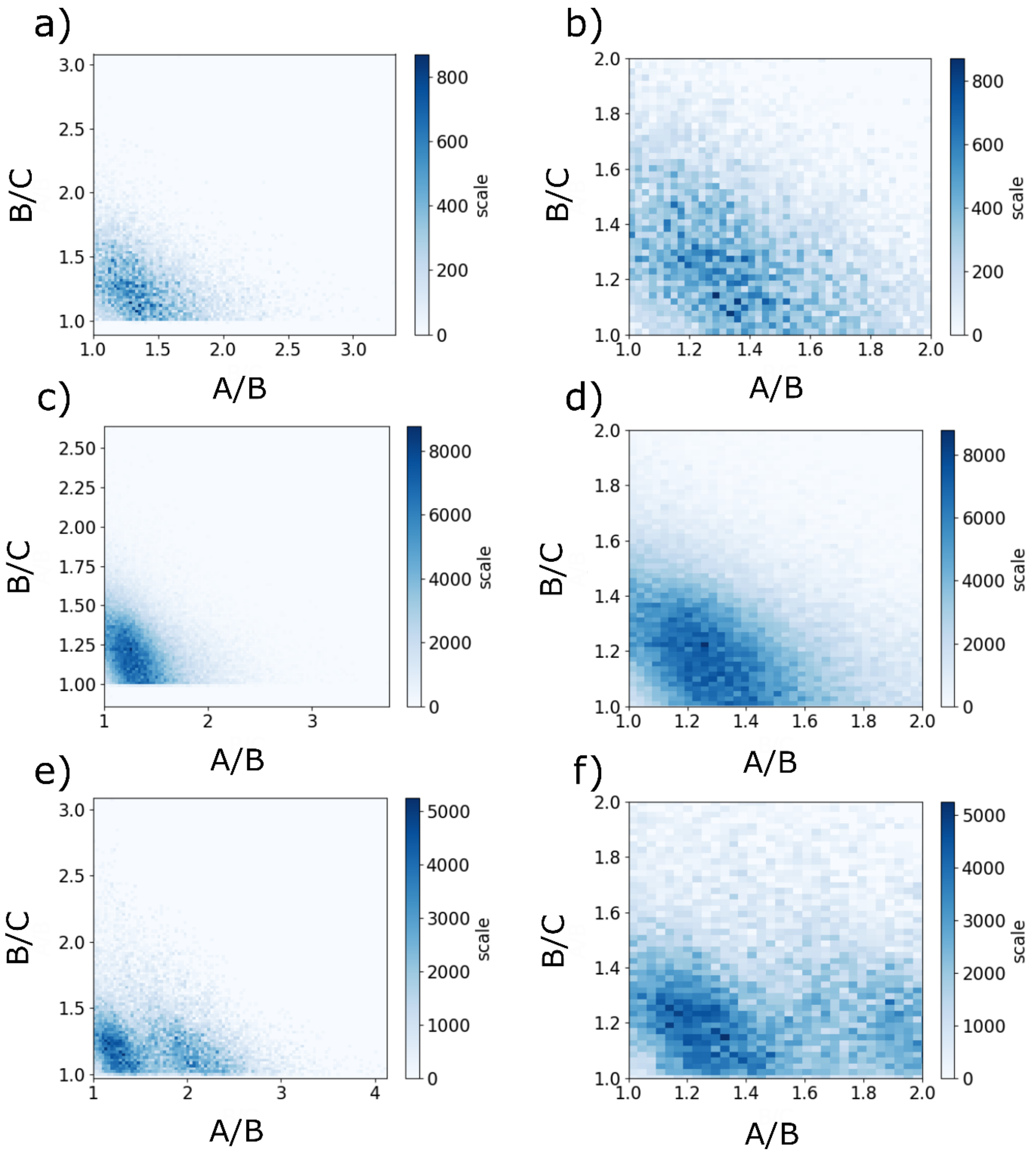

Figure S5: Histograms for DEA systems. a-b) DEA8 histograms. c-d) DEA10 histograms. e-f) DEA12 histograms.

MEGA histograms

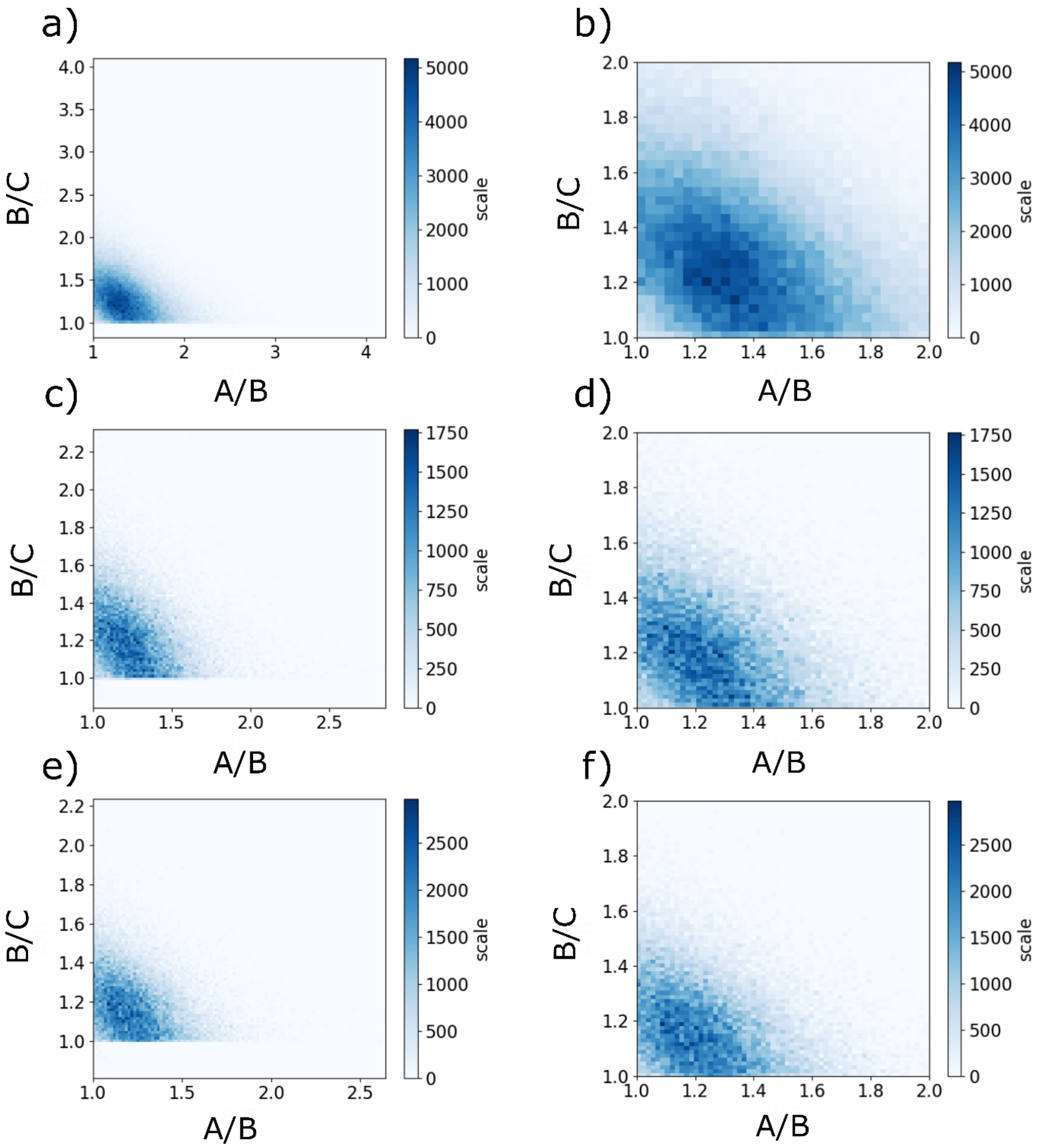

Figure S6: Histograms for MEGA systems. a-b) MEGA8 histograms. c-d) MEGA10 histograms. e-f) MEGA12 histograms.

HEGA histograms

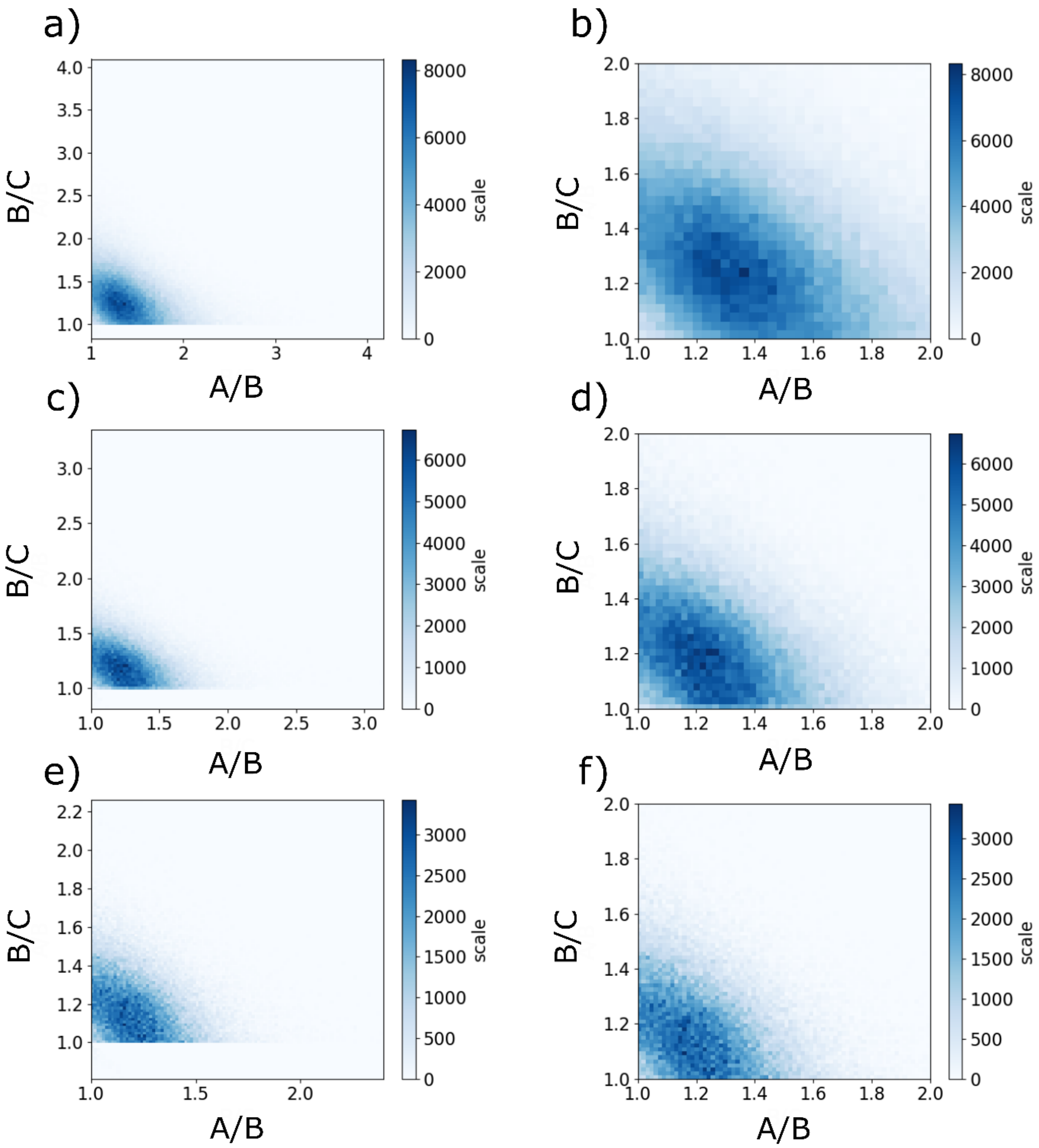

Figure S7: Histograms for HEGA systems. a-b) HEGA8 histograms. c-d) HEGA10 histograms. e-f) HEGA12 histograms.

TEDA, CYGLU and CYMAL histograms

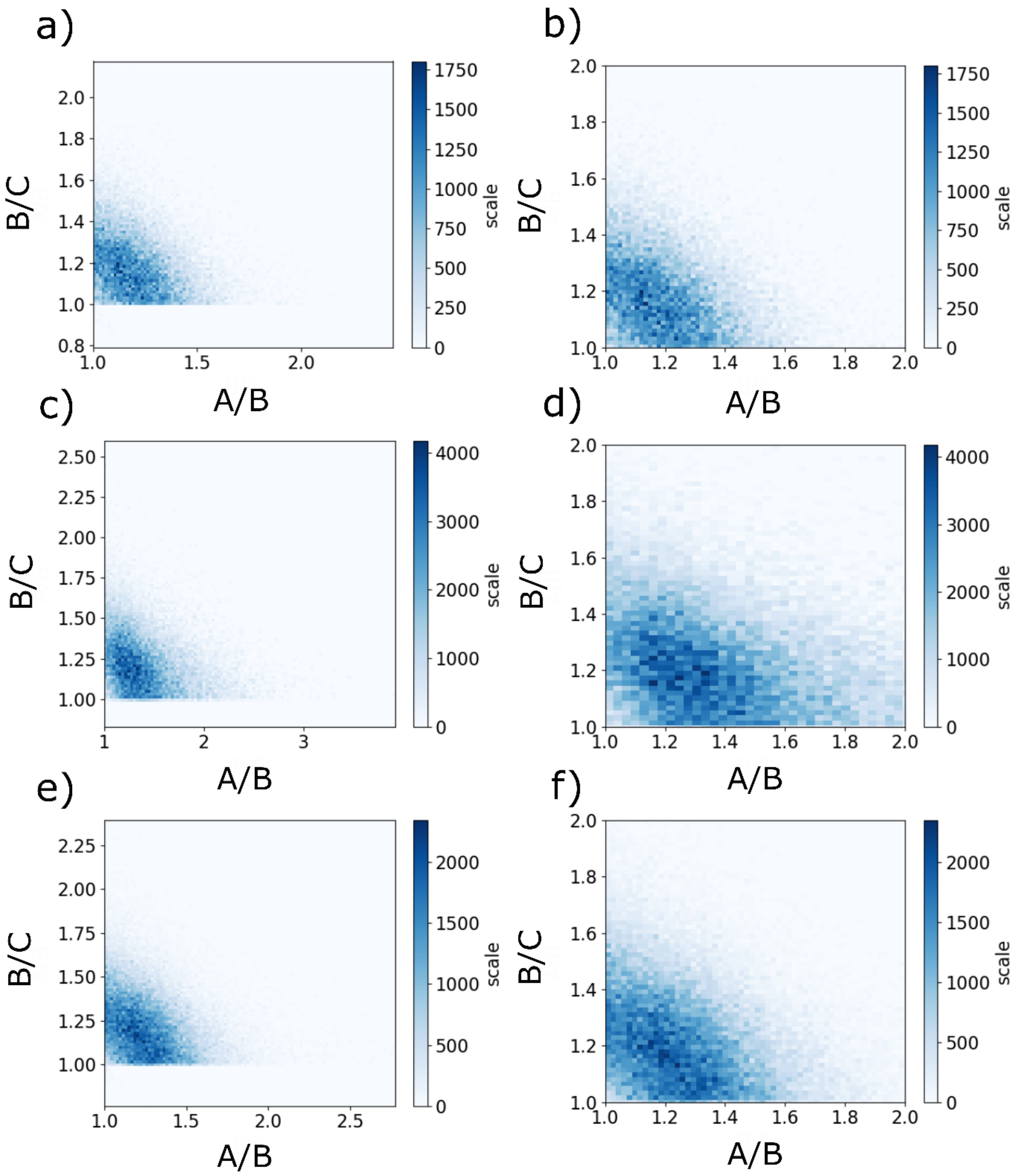

Figure S8: Histograms for TEDA12, CYGLU and CYMAL systems. a-b) TEDA12 histograms. c-d) CYGLU4 histograms. e-f) CYMAL4 histograms.

GLUCO histograms

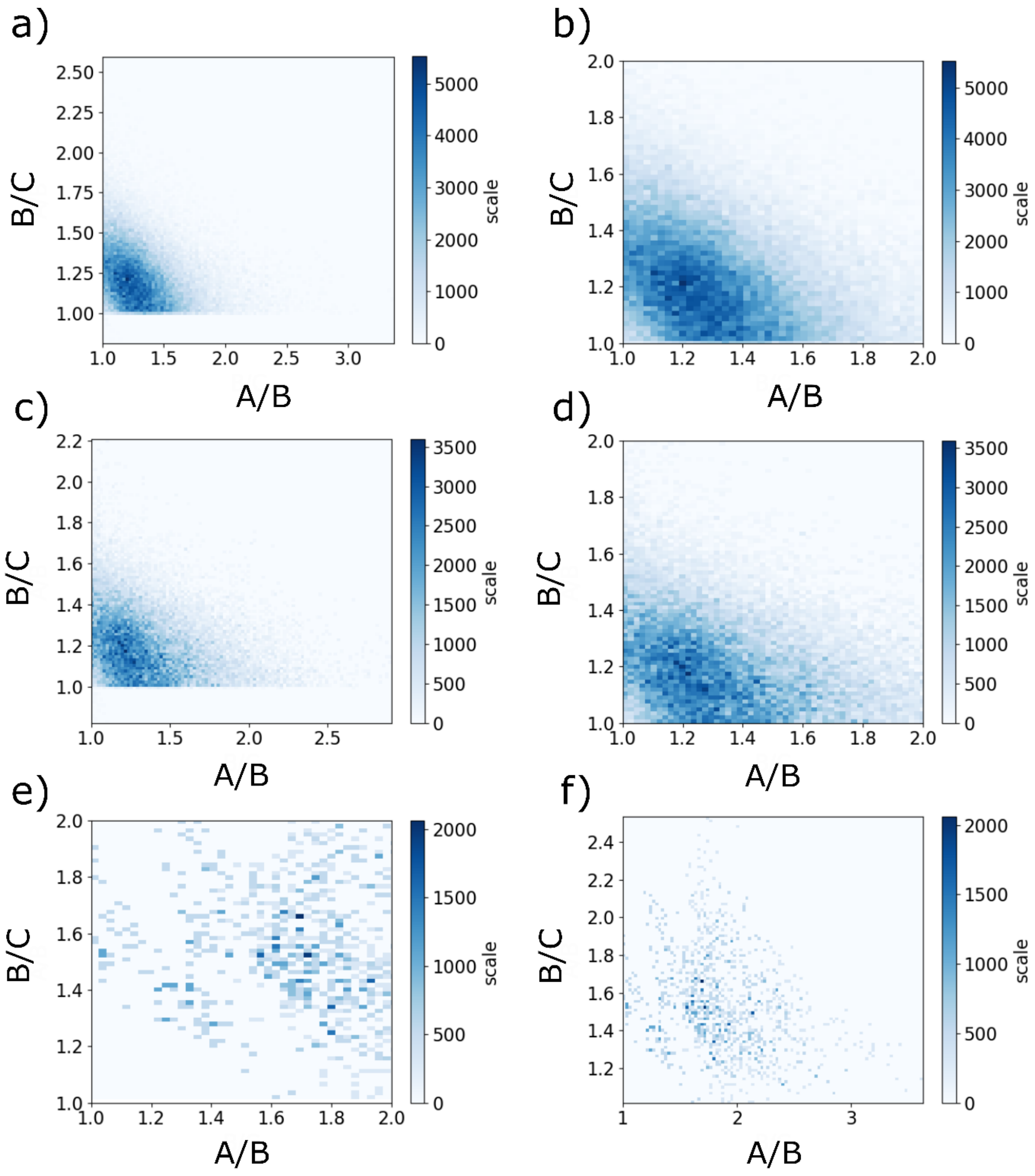

Figure S9: Histograms for GLUCO systems. a-b) GLUCO8 histograms. c-d) GLUCO10 histograms. e-f) GLUCO12 histograms.

MALTO histograms

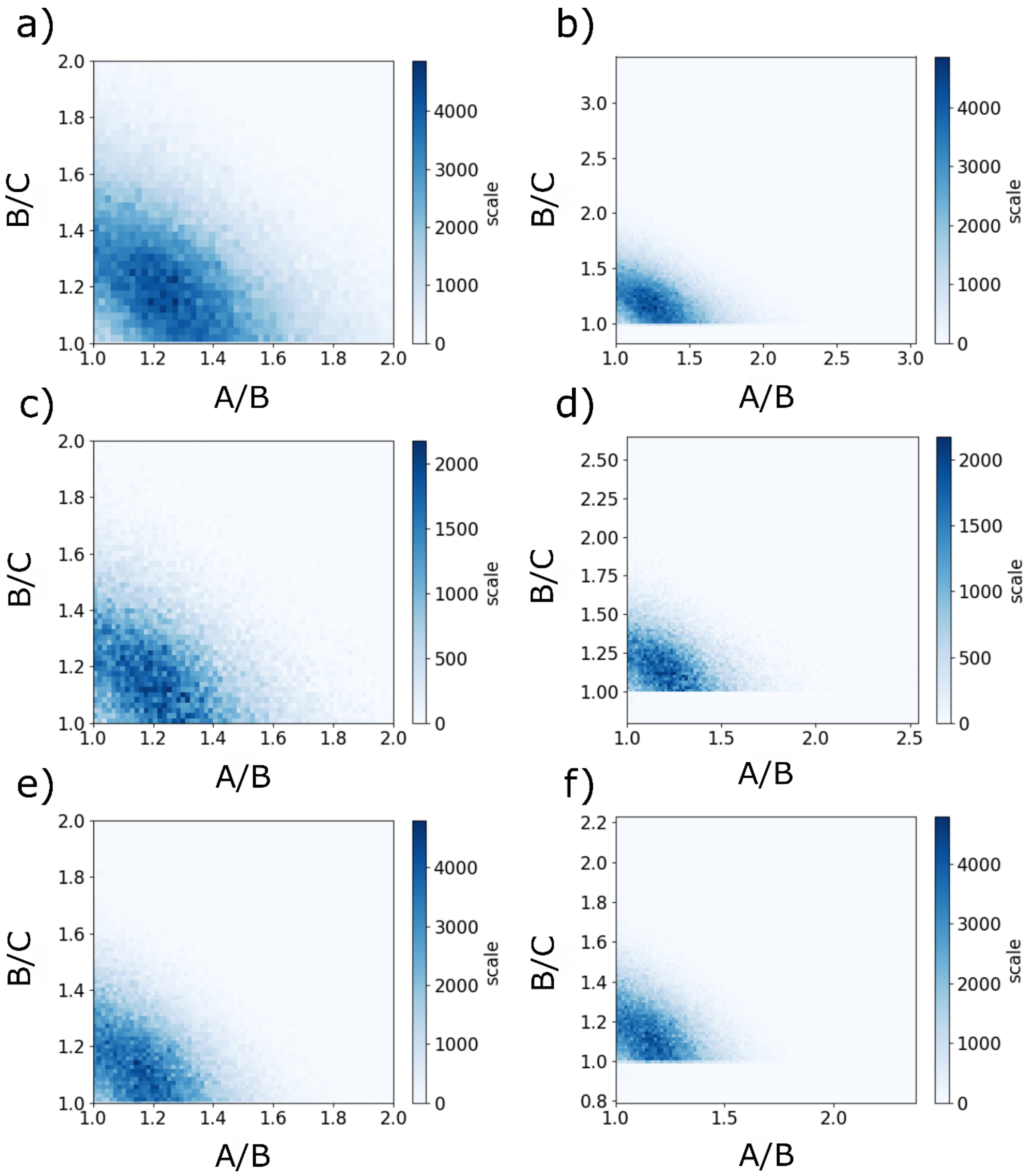

Figure S10: Histograms for MALTO systems. a-b) MALTO8 histograms. c-d) MALTO10 histograms. e-f) MALTO12 histograms.

Parameters for Bonded Interactions

Bead distance calculator script

An open access script was developed to automatise the bead-bead distance calculation. The script can accept either the molecule SMILES, then it performs and show the optimisation or it can use directly an optimised structure in mol2 format. After selecting the molecule fragment used in the bead description and providing the necessary parameters (a_{ij}, R_{ij}, r_{ij} and k_s) the script returns the r_0. The script is freely available and it can be found at:
https://gitlab.developers.cam.ac.uk/el446/bead_distance_calculator.

Bead-bead distance comparison between methods

The reference method consist in the following steps:

- The distances between different fragments were obtained using Molecular Mechanics and translated into DPD units.
- DPD simulations were run with different equilibrium distances and the radial distributions of the two beads were collected.
- The average distance value of the two beads during the DPD simulation was obtained from the weighted average of the radial distribution.
- The values were used as benchmarks for the previous parameterisation method.

A comparison between the two methods is reported in table S1.

Table S1: Fragment distances obtained from MM optimisation, input distance obtained from the recursive (Method 1) and the new method (Method 2) for a selected number of pairwise bead interactions.

Surfactant	Bear pair	MM distance	Method 1: Input distance	Method 2: Input distance	Difference
MEA	AM2 -C2	0.65	0.60	0.58	0.02
MEA	AM2 -OH	0.67/0.68	0.65	0.64/0.65	0.00/0.01
DEA	AM3' -C2	0.67	0.64	0.62	0.02
DEA	AM3' -OH1	0.67	0.66	0.65	0.01
HEGA	AM3' -C2	0.67	0.64	0.62	0.02
HEGA	AM3' -OH1	0.67	0.66	0.65	0.01
HEGA	AM3' -OH'	0.67	0.66	0.65	0.01
MEGA	AM3 -OH'	0.67	0.65	0.65	0.00
MEGA	AM3 -C2	0.68	0.64	0.63	0.01
TEDA	AM2 -EO	0.78	0.75	0.74	0.01
XYL	ES-OH'	0.65	0.63	0.61	0.02
XYL	OH'-OH'	0.44	0.42	0.40	0.02
Tail	C2-C2	0.455	0.39	0.38	0.01
Tail	C2-T	0.35	0.29	0.26	0.03

GLY8 Parameterisation

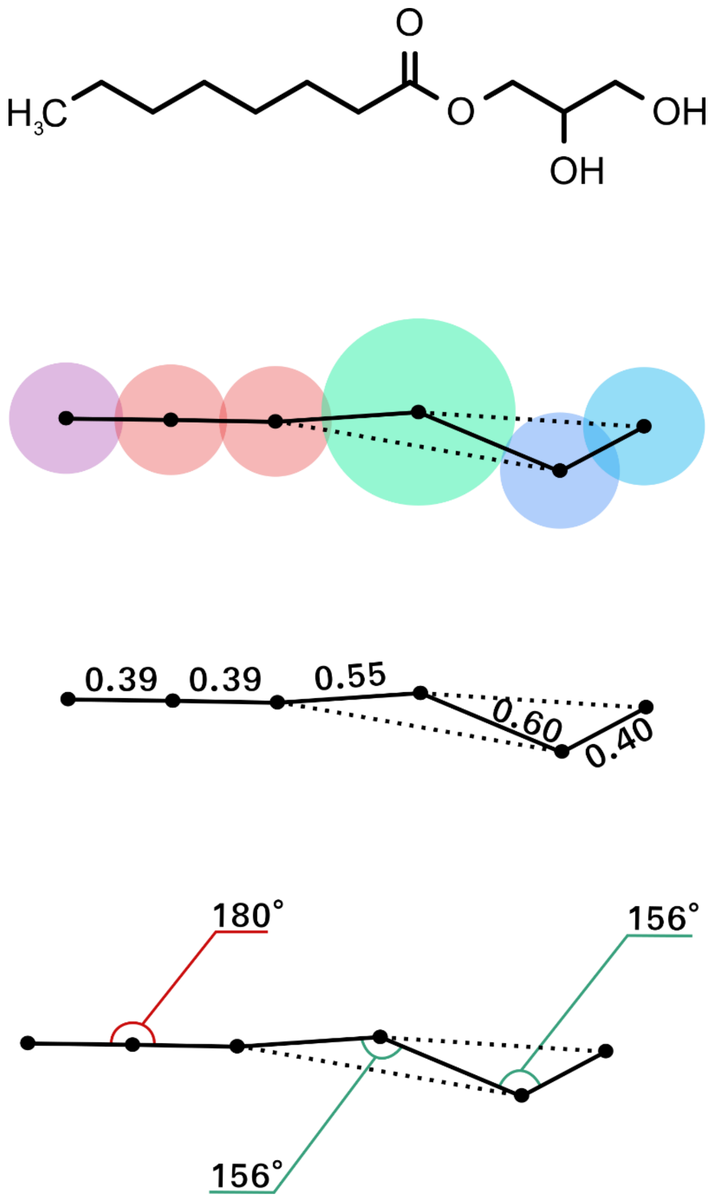

Figure S11: Parameterisation of GLY8: a) shows the 2D surfactant structure, b) shows the GC description with the full lines representing the 1-2 bonds and the dashed lines the 1-3 angles. c) Shows the values for the 1-2 bead distances obtained using equation 11 and and d) shows the 1-3 angles obtained from the MM optimised structure.

XYL8 Parameterisation

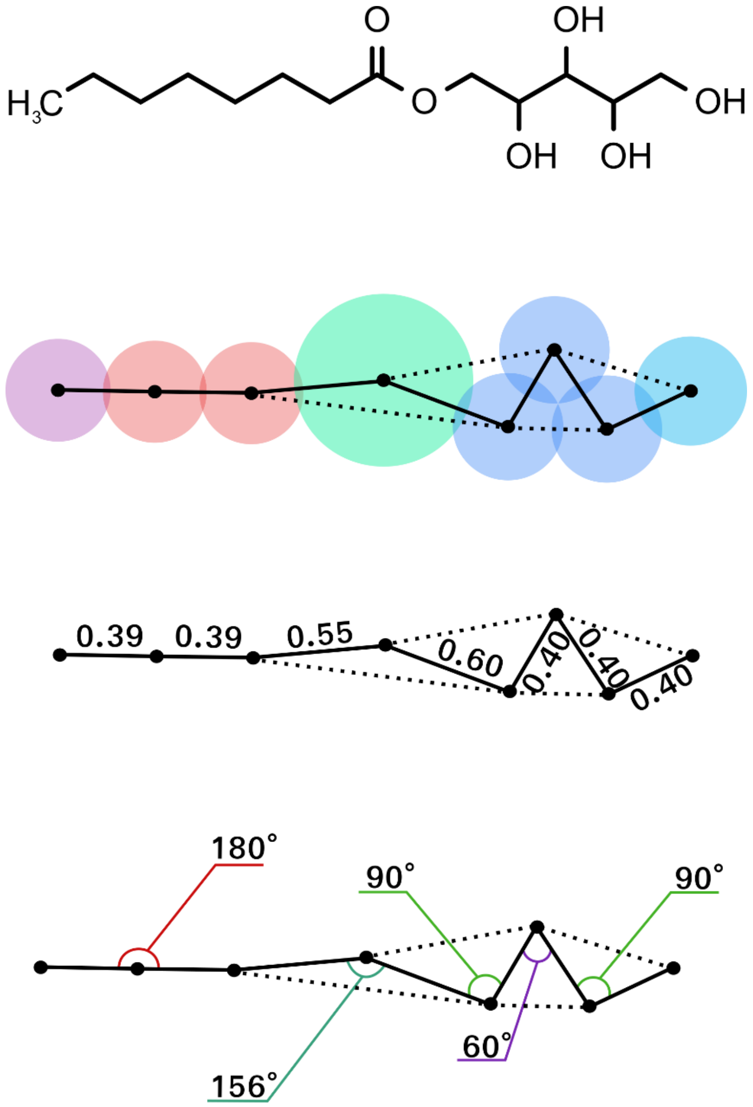

Figure S12: Parameterisation of XYL8: a) shows the 2D surfactant structure, b) shows the GC description with the full lines representing the 1-2 bonds and the dashed lines the 1-3 angles. c) Shows the values for the 1-2 bead distances obtained using equation 11 and d) shows the 1-3 angles obtained from the MM optimised structure.

MEA8 Parameterisation

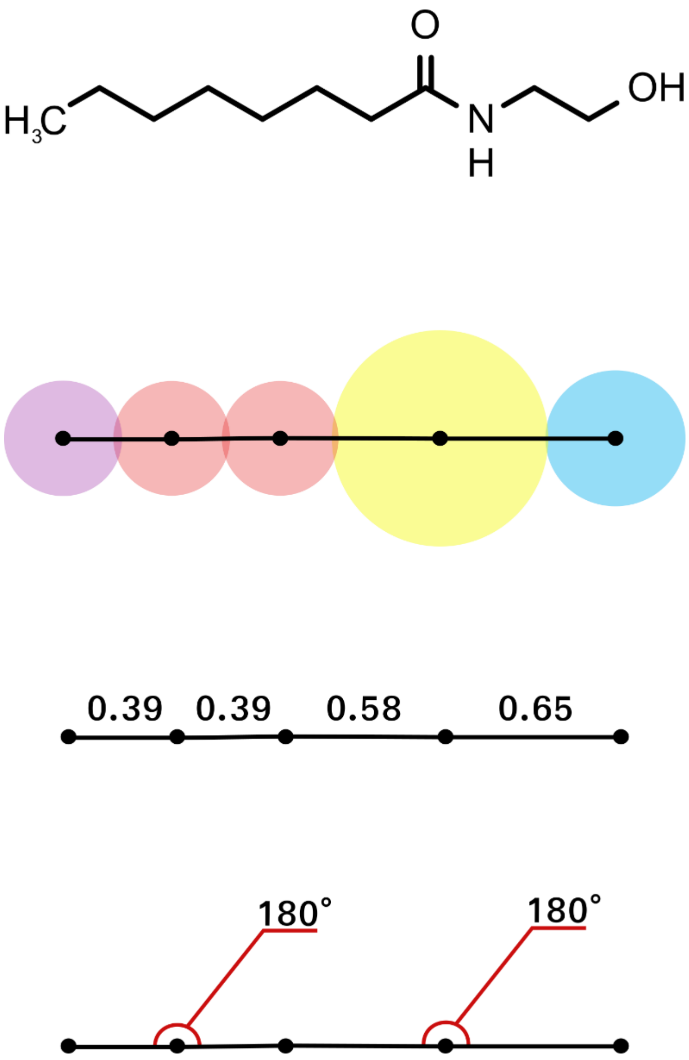

Figure S13: Parameterisation of MEA8: a) shows the 2D surfactant structure, b) shows the GC description with the full lines representing the 1-2 bonds and the dashed lines the 1-3 angles. c) Shows the values for the 1-2 bead distances obtained using equation 11 and d) shows the 1-3 angles obtained from the MM optimised structure.

DEA8 Parameterisation

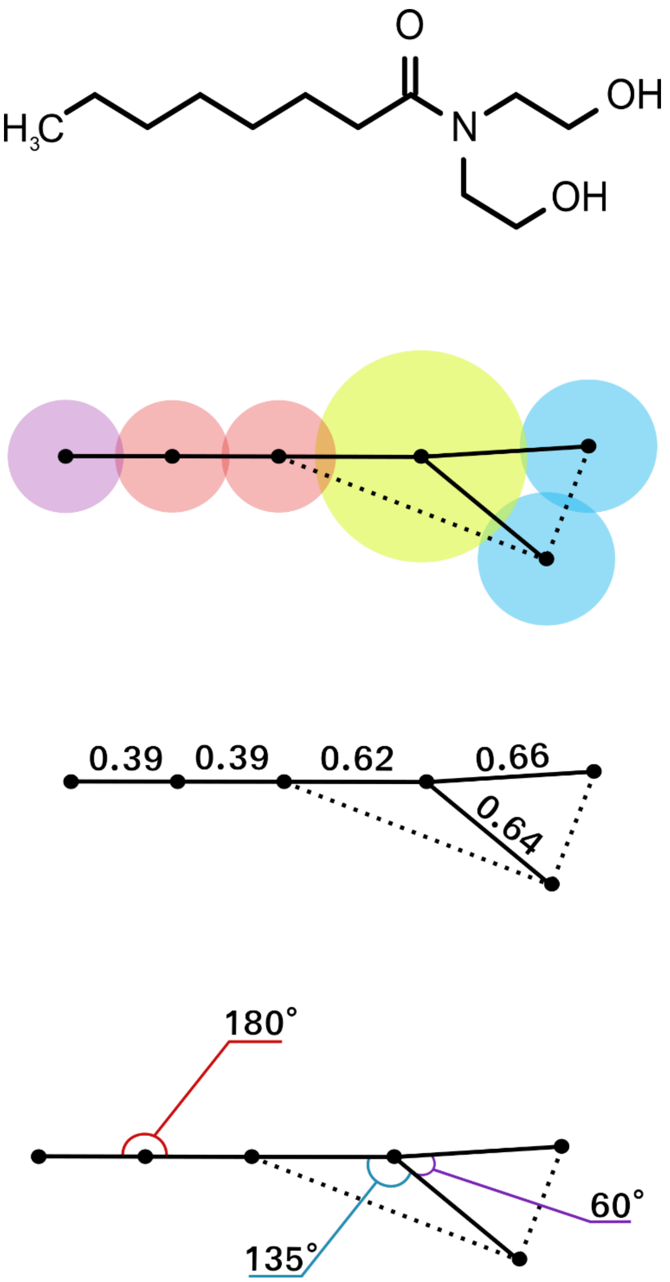

Figure S14: Parameterisation of DEA8: a) shows the 2D surfactant structure, b) shows the GC description with the full lines representing the 1-2 bonds and the dashed lines the 1-3 angles. c) Shows the values for the 1-2 bead distances obtained using equation 11 and d) shows the 1-3 angles obtained from the MM optimised structure.

MEGA8 Parameterisation

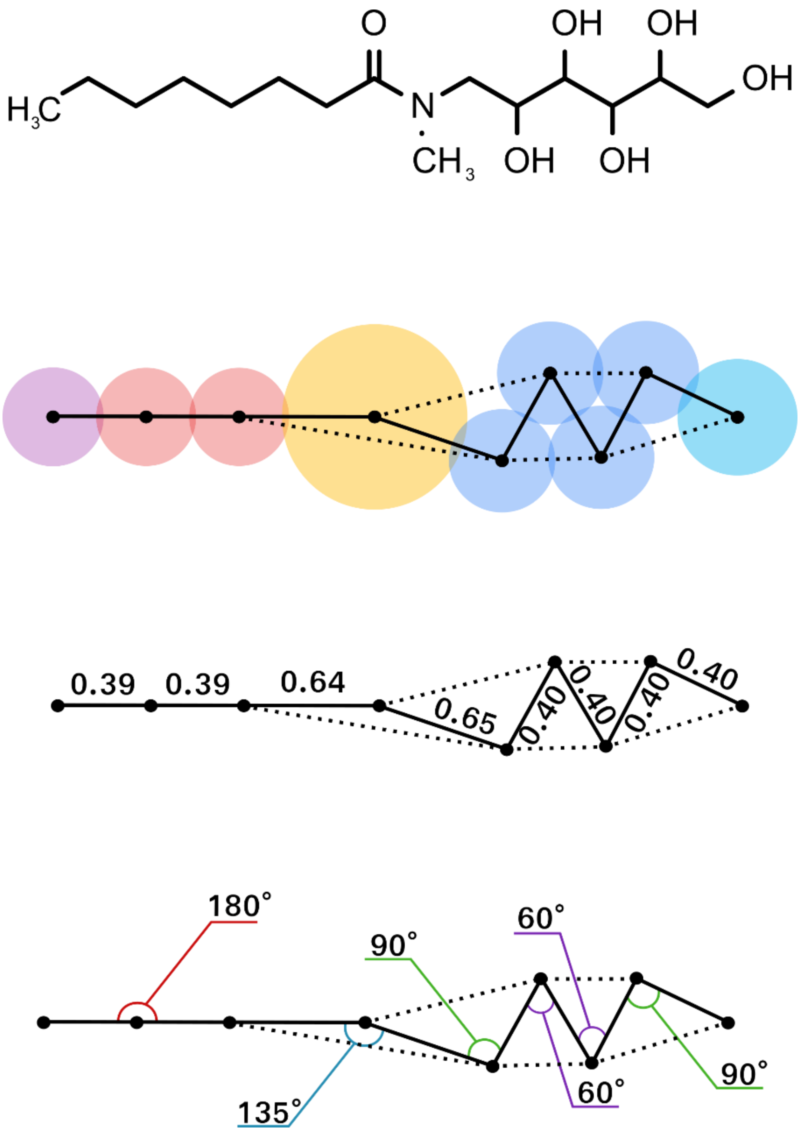

Figure S15: Parameterisation of MEGA8: a) shows the 2D surfactant structure, b) shows the GC description with the full lines representing the 1-2 bonds and the dashed lines the 1-3 angles. c) Shows the values for the 1-2 bead distances obtained using equation 11 and d) shows the 1-3 angles obtained from the MM optimised structure.

HEGA8 Parameterisation

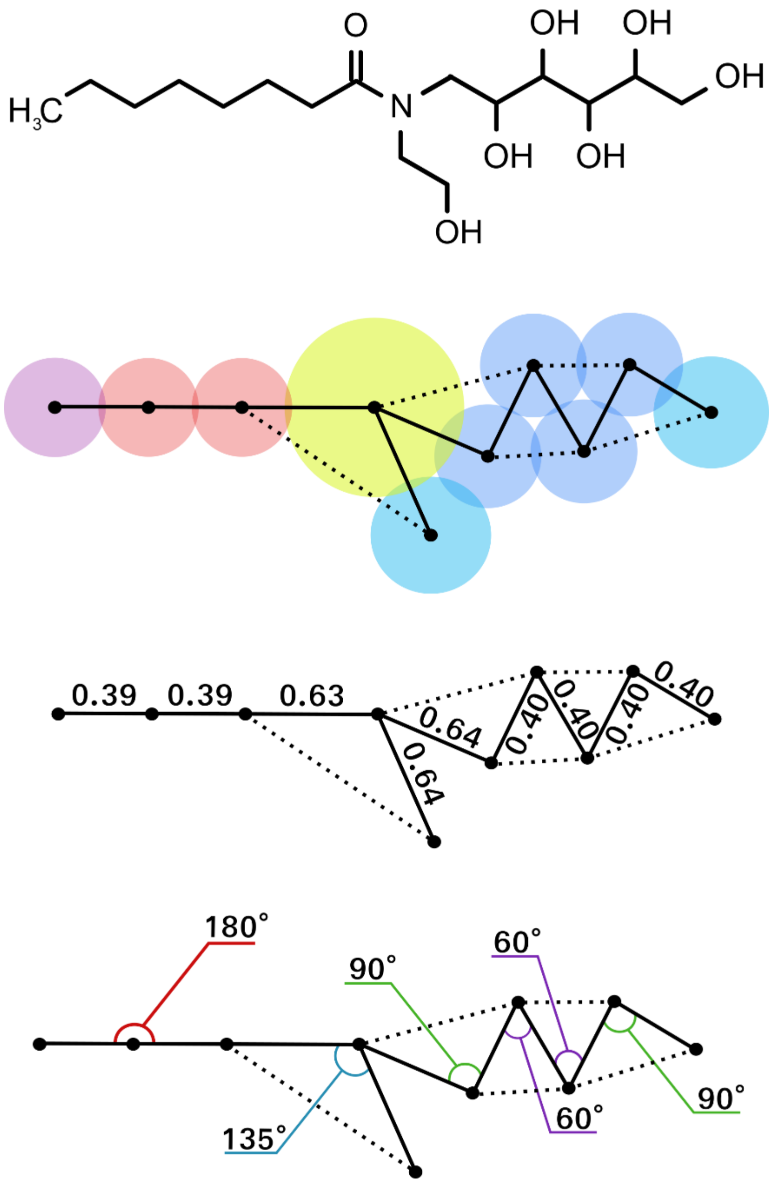

Figure S16: Parameterisation of HEGA8: a) shows the 2D surfactant structure, b) shows the GC description with the full lines representing the 1-2 bonds and the dashed lines the 1-3 angles. c) Shows the values for the 1-2 bead distances obtained using equation 11 and d) shows the 1-3 angles obtained from the MM optimised structure.

TEDA12 Parameterisation

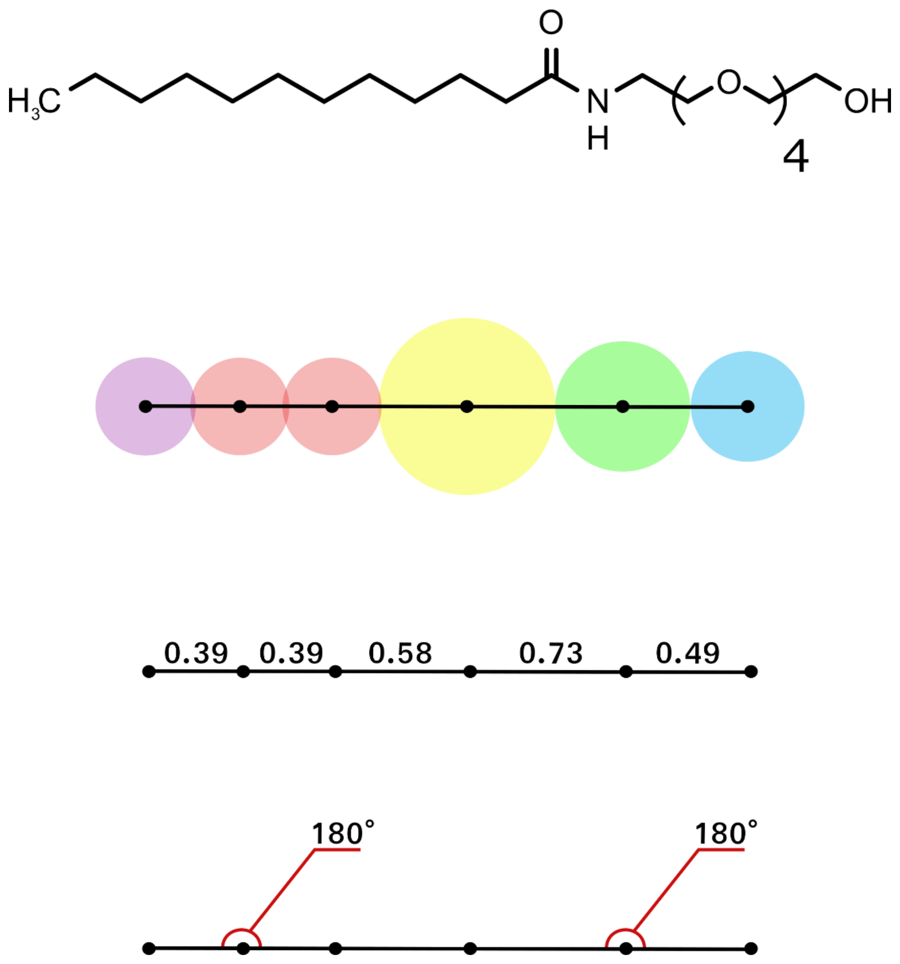

Figure S17: Parameterisation of TEDA12: a) shows the 2D surfactant structure, b) shows the GC description with the full lines representing the 1-2 bonds and the dashed lines the 1-3 angles. c) Shows the values for the 1-2 bead distances obtained using equation 11 and d) shows the 1-3 angles obtained from the MM optimised structure.

CYGLU4 Parameterisation

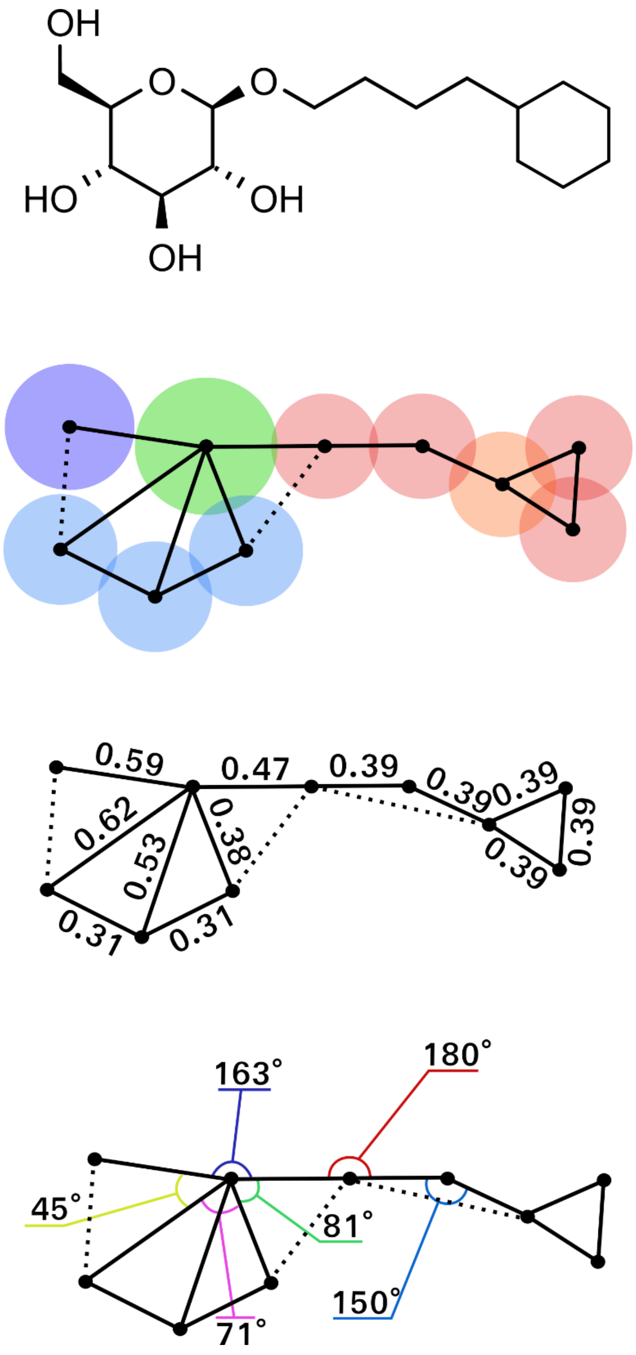

Figure S18: Parameterisation of CYGLU4: a) shows the 2D surfactant structure, b) shows the GC description with the full lines representing the 1-2 bonds and the dashed lines the 1-3 angles. c) Shows the values for the 1-2 bead distances obtained using equation 11 and d) shows the 1-3 angles obtained from the MM optimised structure.

CYMAL4 Parameterisation

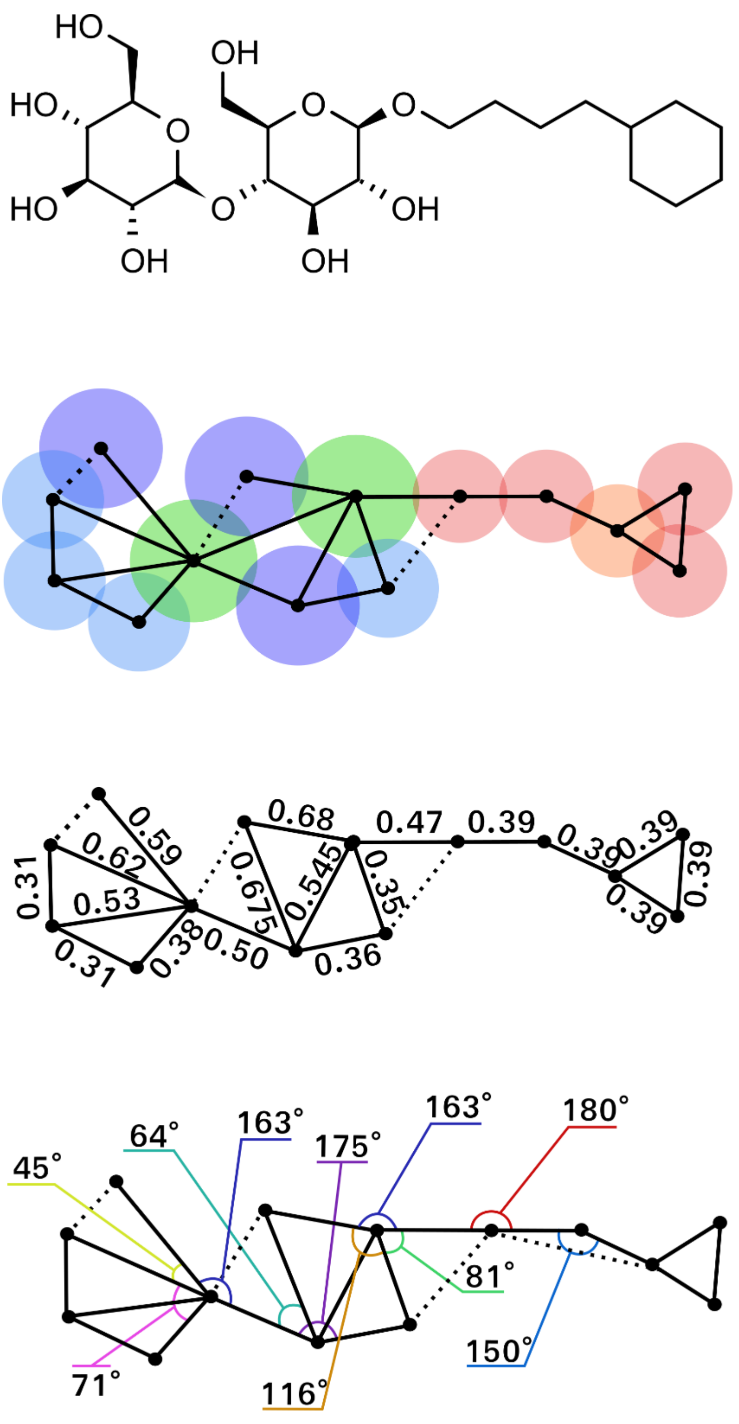

Figure S19: Parameterisation of CYMAL4: a) shows the 2D surfactant structure, b) shows the GC description with the full lines representing the 1-2 bonds and the dashed lines the 1-3 angles. c) Shows the values for the 1-2 bead distances obtained using equation 11 and d) shows the 1-3 angles obtained from the MM optimised structure.

GLUCO8 Parameterisation

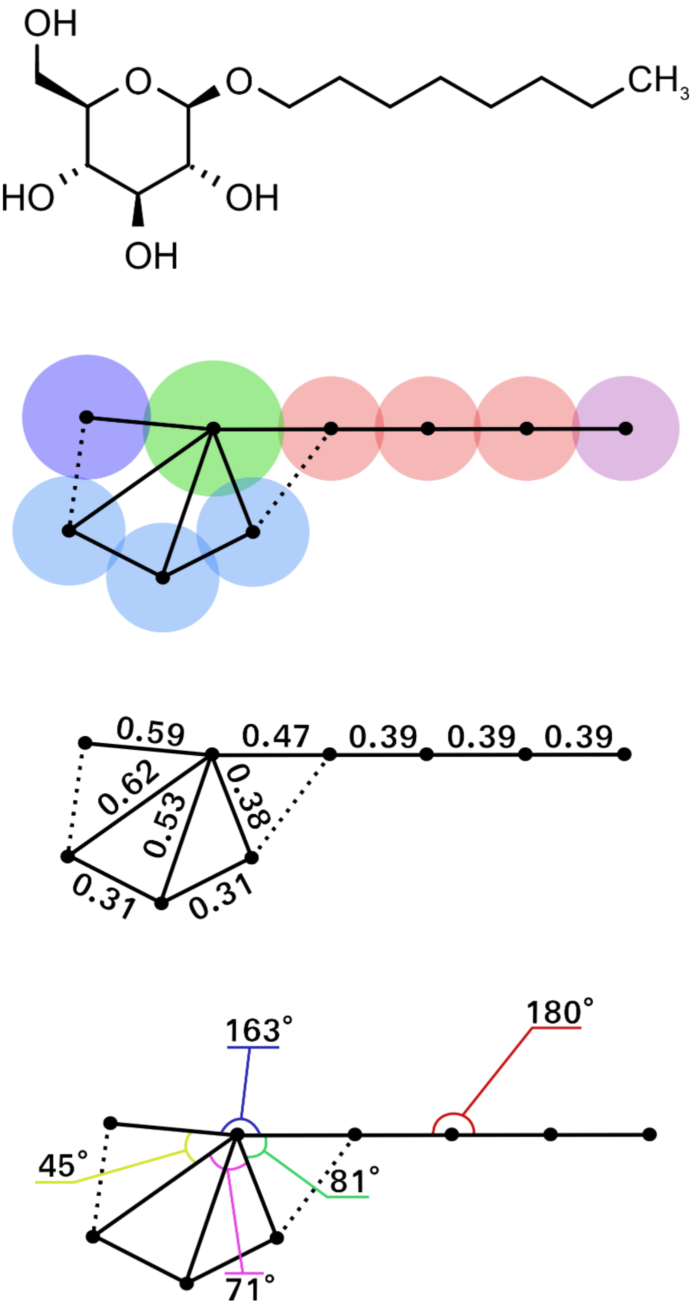

Figure S20: Parameterisation of GLU8: a) shows the 2D surfactant structure, b) shows the GC description with the full lines representing the 1-2 bonds and the dashed lines the 1-3 angles. c) Shows the values for the 1-2 bead distances obtained using equation 11 and d) shows the 1-3 angles obtained from the MM optimised structure.

MALTO8 Parameterisation

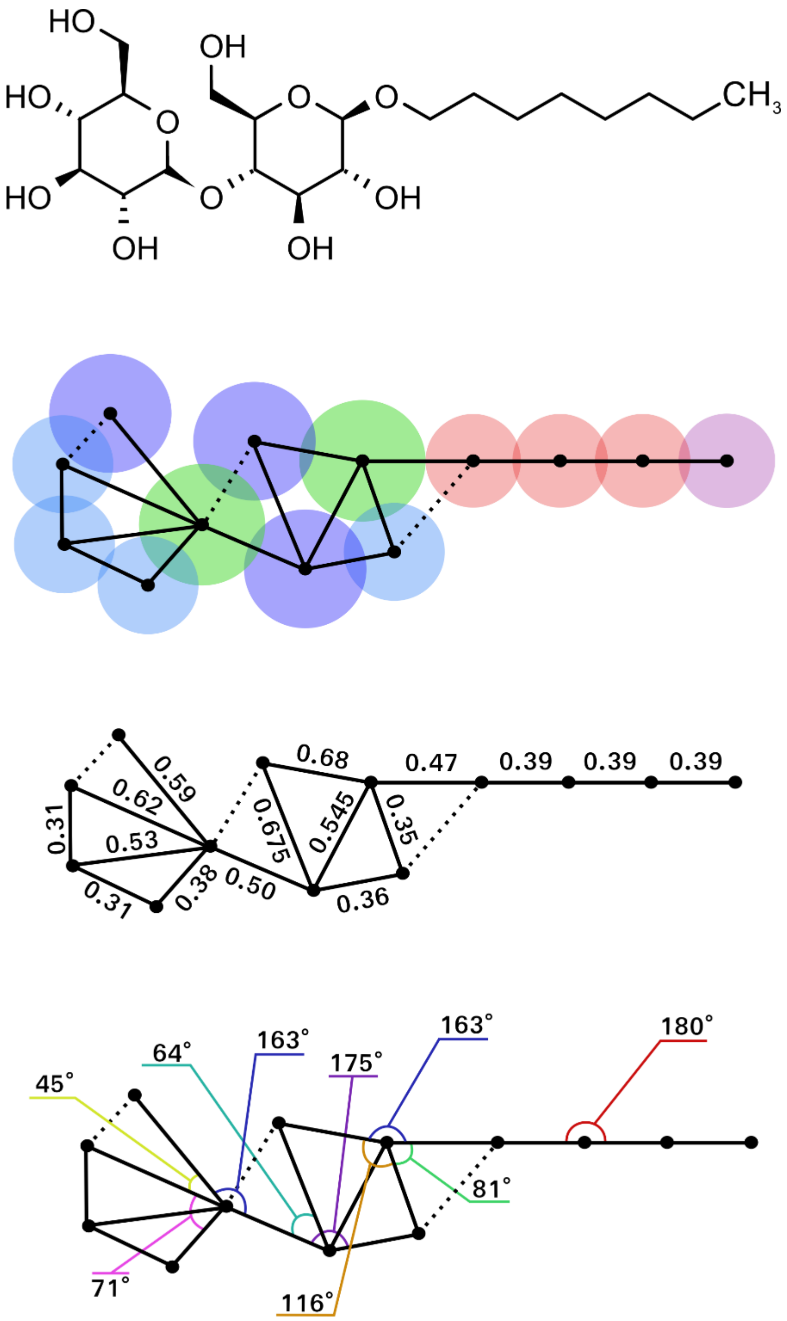

Figure S21: Parameterisation of MAL8: a) shows the 2D surfactant structure, b) shows the GC description with the full lines representing the 1-2 bonds and the dashed lines the 1-3 angles. c) Shows the values for the 1-2 bead distances obtained using equation 11 and d) shows the 1-3 angles obtained from the MM optimised structure.

Amide Conformers

Table S2: Calculated and experimental values of CMC and N_{agg}.

Surfactant	Amide	CMC/mM calc.	N_{agg} calc.	CMC/mM exp.	N_{agg} exp.
MEGA8	*trans*	101.03±3.56	9-18	51-79	24-85
	cis	112.32±4.26	9-12		
MEGA10	*trans*	9.44±0.45	26-28	4-7	28-75
	cis	11.03±0.52	24-27		
MEGA12	*trans*	1.02±0.11	26-36	0.35	-
	cis	1.32±0.24	22-25		
